# Supplementary material for: Taxonomy, Phylogeny, Divergence Time Estimation, and Biogeography of the Family Pseudoplagiostomataceae (Ascomycota, Diaporthales)
Source: J Fungi (Basel). 2023 Jan 5;9(1):82. doi: 10.3390/jof9010082 (PMC9860658; doi:10.3390/jof9010082)
Supplement: Supplementary file 1 [file jof-09-00082-s001.zip › jof-2120314-Supplementary File S1.pdf]

>Apoharknessia_insueta_CBS_111377
CGAGTGAAGCGGCAACAGCTCAAATTTGAAATCTGGCCTCGT-
GCCCGAGTTGTAATTTGCAGAGGATGTTTCTGGCGCGGTGCCGTCCGAGTTTCCTGGAACGGAACGCCAA
AGAGGGTGAGAGCCCCGTATGGTCGGACACCAAGCCTGTGTGAAACTCCTTCGACGAGTCGAGTAGTTTG
GGAATGCTGCTCTAAATGGGAGGTAAATCTCTTCTAAAGCTAAATACCGGCCAGAGACCGATAGCGCACA
AGTAGAGTGATCGAAAGATGAAAAGCACCTTGAAAAGGGGGTTAAACAGTACGTGAAATTGTTGAAAGGG
AAGCGTTTGTGACCAGACTTGTGCCAGGCGGCTCATCCGGCGTTCTCGCCGGTGCACTCCGCCTGTGCTC
AGGCCAGCATCGGTTCTCGTGGGGGGAGAAGAGCGGTGGGAATGTGGCTCACCCTCGGGTGAGTGTTATA
GCCCGCCGCACGATGCCCCCGTGGGGACCGAGGTTCGCGCTTCC----
GCATGGATGCTGGCGTAATGGTCACTAGCGACCCGTCTTGAAACACGGACCAAGGAGTCGTCCATTAGAG
CGAGCGTTTGGGTGTCAAACCCGCACGCGTAATGAAAGTGAAAT-----
TAGGTGAGAGCTTCGGCGCATCATCGACCGATCCTGATGTTCTCGGATGGATTTGAGTAAGAGTTTTAAC
GGACGGACCCGAAAGACAGTGAACTATGCTTGTATAGGGTGAAGCCAGAGGAAACTCTGGTGGAGGCTCG
CAGCGGTTCTGACGTGCAAATCGATCGTCAAATATGAGCATGGGGGCGAAAGACTAATCGAACTGTCTAG
TA---------------------AGGGATCATTGCTGGAA-GCGTCGCAAGACGCGCCCAG---
ATTACCCCTATGTGAACCTTTATT--
TTATCGTTGCCTCGGTCAAGGCTGGGTCTCCTCGCAGACCCCCTTCTCCTGACGTCCTCCTCCTCT--
GGAGGGGAGACGGCAGGGAGGGAGCAGCCGGCCGTTGGCCC-----TTATAACTCTTG-
TCTTTATCTGG---AATTCTTCTGAGTA-AAACAAAC---
AAATGAATCAAAACTTTCAACAACGGATCTCTTGGTTCTGGCATCGATGAAGAACGCAGCGAAATGCGAT
AAGTAATGTGAATTGCAGAATTCAGTGAATCATCGAATCTTTGAACGCACATTGCGCCCGCTGGTATTCC
AGCGGGCATGCCTGTTCGAGCGTCATTTCAACCCTC----AAGCCCAG----------------------
---CTTGGTGTTGGGACATT--GCC---------CTCCTCGTCGA------------------
GGGCAAGTCCTTAAATTGAGTGGCGAGCTCGCCAGGA---CTCCGAGCGTAGTAGTTT--------
TACCACCTCGCTT--TGGACGGACTGGCGGTGCCTCGGCCGTAAAA--CCCCCCAATTTATTAC-
CAAAGGTGACCTCGGATCAGGTAGGAATACCCGCTGAACTTAA---------------------------
----

--TGCTGATTCCACA----
CGGACATTTGCAT----------GATTGCGAACGAGTT------
GCTATCTGCTTTGCAGTCGTTTGTCGGCCTCCCTTTTTGCCCTGTCCCACCACAT-
TTTCGCTTTTTTGGTGCGGGG-TTGCGTGGGTTCTTATCTTTAGATAAACGTGACCCGCT-----
CAACCCCAGCCAGAG-
AATTCTTACTATGGCCCTTCGCCACGTATACACAGACCCCCTCCCCAACGCCGTC----
CATCGTCGCCGCCATAATGACATCGTGCTAACTC-AATT-
CGACCATAGCCGCCGAGCTCGGTAAGGGTTCCTTCAAGTACGCCTGGGTCCTTGACAAGCTCAAGGCCGA
GCGTGAGCGTGGTATCACCATCGACATTGCTCTGTGGAAGTTCGAGACTCCCAAGTACTATGTCACCGTC

ATTGGTATGTTTTATGTTC-
CGTGGCTTTTGCAAGCCGACATGCAAGCTTGACAACTGTCCAATTCCAACCCATTGTACTAATGACAACG
CCAA--

>Apoharknessia_insueta_CBS_114575
CGAGTGAAGCGGCAACAGCTCAAATTTGAAATCTGGCCTCG--
GCCCGAGTTGTAATTTGCAGAGGATGTTTCTGGCGCGGTGCCGTCCGAGTTTCCTGGAACGGAACGCCAA
AGAGGGTGAGAGCCCCGTATGGTCGGACACCAAGCCTGTGTGAAACTCCTTCGACGAGTCGAGTAGTTTG
GGAATGCTGCTCTAAATGGGAGGTAAATCTCTTCTAAAGCTAAATACCGGCCAGAGACCGATAGCGCACA
AGTAGAGTGATCGAAAGATGAAAAGCACCTTGAAAAGGGGGTTAAACAGTACGTGAAATTGTTGAAAGGG
AAGCGTTTGTGACCAGACTTGTGCCAGGCGGCTCATCCGGCGTTCTCGCCGGTGCACTCCGCCTGTGCTC
AGGCCAGCATCGGTTCTTGTGGGGGGAGAAGAGCGGTGGGAATGTGGCTCACCCTCGGGTGAGTGTTATA
GCCCGCCGCACGATGCCCCCGTGGGGACCGAGGTTCGCGCTTCC----
GCATGGATGCTGGCGTAATGGTCACTAGCGACCCGTCTTGAAACACGGACCAAGGAGTCGTCCATTAGAG
CGAGCGTTTGGGTGTCAAACCCGCACGCGTAATGAAAGTGAAAT-----
TAGGTGAGAGCTTCGGCGCATCATCGACCGATCCTGATGTTCTCGGATGGATTTGAGTAAGAGTTTTAAC
GGACGGACCCGAAAGACAGTGAACTATGCTTGTATAGGGTGAAGCCAGAGGAAACTCTGGTGGAGGCTCG
CAGCGGTTCTGACGTGCAAATCGATCGTCAAATATGAGCAT-GGTGCGAAAGACTAATCG----------
---------------------------ATCATTGCTGGAA-GCGTCGCAAGACGCGCCCAG---
ATTACCCCTATGTGAACCTTTATT--
TTATCGTTGCCTCGGTCAAGGCTGGGTCTCCTCGCAGACCCCCTTCTCCTGACGTCCTCCTCCTCT--
GGAGGGGAGACGGCAGGGAGGGAGCAGCCGGCCGTTGGCCC-----TTATAACTCTTG-
TCTTTATCTGG---AATTCTTCTGAGTA-AAACAAAC---
AAATGAATCAAAACTTTCAACAACGGATCTCTTGGTTCTGGCATCGATGAAGAACGCAGCGAAATGCGAT
AAGTAATGTGAATTGCAGAATTCAGTGAATCATCGAATCTTTGAACGCACATTGCGCCCGCTGGTATTCC
AGCGGGCATGCCTGTTCGAGCGTCATTTCAACCCTC----AAGCCCAG----------------------
---CTTGGTGTTGGGACATT--GCC---------CTCCTCGTCGA------------------
GGGCAAGTCCTTAAATTGAGTGGCGAGCTCGCCAGGA---CTCCGAGCGTAGTAGTTT--------
TACCACCTCGCTT--TGGACGGACTGGCGGTGCCTCGGCCGTAAAA--CCCCCCAATTTATTAC-
CAAAGGTGACCTCGGATCAGGTAGGAATACCCGCTGAACTTAA---------------------------
----

--------------GAGGGTATGTATTCATCTGTTCCTAT---ATTCTGATTCTCCCAA--
GGGACATTCGTGT----------GATTGCGAACG-------
GTTGCGACCTGCTTTGCAGTCGTTTGTCGGCCTCCCTTTTTGCCCTGTCCCACCACAT-
TTTCGCTTTTTTGGTGCGGGG-TTGCGTGGGTTCTTATCTTG-GATAAACGTGACCCGCT-----
CAACCCCAGCCAGAGAATTCATTACTATGGCCCTCCKCCATGTACACACAGACCCCCTCCCCAACGCCGT
C----AATCGTCGTCGCTACAATGACATCGTGCTAACTT-
AATTTGCACCATAGCCGCCGAGCTCGGTAAGGGTTCCTTCAAGTACGCCTGGGTCCTTGACAAGCTCAAG
GCCGAGCGTGAGCGTGGTATCACCATCGACATTGCTCTGTGGAAGTTCGAGACTCCCAAGTACTATGTCA
CCGTCATTGGTATGTTCTATGCTTCCGTGGCTTTTGCAAGCCGACATGCAAGCTTGACAACTGTCCAATT
TCAACGCATTGTACTAATGACAACGCCATAGACGCTCCCGGTCACCGTGACTTCATCAAGAACATG----
----

>Apoharknessia_eucalypti_CBS_142518
CGAGTGAAGCGGCAACAGCTCAAATTTGAAATCTGGCCTCGT-
GCCCGAGTTGTAATTTGCAGAGGATGTTTCTGGCGCGGTGCCGTCCGAGTTTCCTGGAACGGAACGCCAA
AGAGGGTGAGAGCCCCGTATGGTCGGACACCAAGCCTGTGTGAAACTCCTTCGACGAGTCGAGTAGTTTG
GGAATGCTGCTCTAAATGGGAGGTAAATCTCTTCTAAAGCTAAATACCGGCCAGAGACCGATAGCGCACA
AGTAGAGTGATCGAAAGATGAAAAGCACCTTGAAAAGGGGGTTAAACAGTACGTGAAATTGTTGAAAGGG
AAGCGTTTGTGACCAGACTTGTGCCAGGCGGCTCATCCGGCGTTCTCGCCGGTGCACTCCGCCTGTGCTC
AGGCCAGCATCGGTTCTCGTGGGGGGAGAAGAGCGATGGGAATGTGGCTCACCCTCGGGTGAGTGTTATA
GCCCGCCGCACGATGCCCCCGTGGGGACCGAGGTTCGCGCTTCC----
GCATGGATGCTGGCGTAATGGTCACTAGCGACCCGTCTTGAAACACGGACCAAGGAGTCGTCCATTAGAG
CGAGCGTTTGGGTGTCAAACCCGCACGCGTAATGAAAGTGAAAT-----
TAGGTGAGAGCTTCGGCGCATCATCGACCGATCCTGATGTTCTCGGATGGATTTGAGTAAGAGTTTTAAC
GGACGGACCCGAAAGACAGTGAACTATGCTTGTATAGGGTGAAGCCAGAGGAAACTCTGGTGGAGGCTCG
CAGCGGTTCTGACGTGCAAATCGATCGTCAAATATGAGCATGGGGGCGAAAGACTAATCG----------
--------------AACCAGCGGAGGGATCATTGCTGGAA-GCGTCGCAAGACGCGCCCAG---
ATCACCCCTATGTGAACTTATA----
TTATCGTTGCCTCGGTCAAGGCTGGGTCTCTCTGAGATCCCCTCTCTGCGGTCCTCTCCTTCCTTCGGGA
AGGGAGGGACGACAGGAGGAG-CCGCCGGCCGTTGGCCC-----TTTACACTCTTG-TCTCCATCTGG--
-ATTTCTTCTGAGTATAAACAAAC---
AAATGAATCAAAACTTTCAACAACGGATCTCTTGGTTCTGGCATCGATGAAGAACGCAGCGAAATGCGAT
AAGTAATGTGAATTGCAGAATTCAGTGAATCATCGAATCTTTGAACGCACATTGCGCCCGCTGGTATTCC
AGCGGGCATGCCTGTTCGAGCGTCATTTCAACCCTC----AAGCCCAG----------------------
---CTTGGTGTTGGGACATT--GCCA--TCTTCGTCCATCTTCTGGATGCACGA---------
GGGCAAGTCCTCAAATTGAGTGGCGAGCTCGCCAGGA---CTCCGAGCGTAGTAGTTT--------

TACCACCTCGCTT--TGGACGGACTGGCGGTGCCTCAGCCGTAAAA--
CCCCCCAATTTATTACCAAAGGTTGACCTCGGATCAGGTAGGAATACCCGCTGAACTTAA----------
----

CCTTCTGGTGTGTACACACAGCTTCACGGCCACACCGCGTCGATCGGGCACGGCGGTGTTTTGGTATCGT
GACATTGTTACTGACCTCAATTGCAGGCAAACCATCTCTGGCGAGCACGGCCTCGACAGCAATGGTGTGT
ACGTG-------TCAACCCTCCATACCTTCTTGACACTTGTCT--GC--CTTCGAGACTGACCT-
CGCCGCAGTTACAACGGCACCTCCGAGCTGCAGCTCGAGCGCATGAACGTCTACTTCAACGAGGTATGTC
GA--

>Apoharknessia_eucalyptorum_CBS_142519
CGAGTGAAGCGGCAACAGCTCAAATTTGAAATCTGGCCTCGT-
GCCCGAGTTGTAATTTGCAGAGGATGTTTCTGGCGCGGTGCCGTCCGAGTTTCCTGGAACGGAACGCCAA
AGAGGGTGAGAGCCCCGTATGGTCGGACACCAAGCCTGTGTGAAACTCCTTCGACGAGTCGAGTAGTTTG
GGAATGCTGCTCTAAATGGGAGGTAAATCTCTTCTAAAGCTAAATACCGGCCAGAGACCGATAGCGCACA
AGTAGAGTGATCGAAAGATGAAAAGCACCTTGAAAAGGGGGTTAAACAGTACGTGAAATTGTTGAAAGGG
AAGCGTTTGTGACCAGACTTGTGCCAGGCGGCTCATCCGGCGTTCTCGCCGGTGCACTCCGCCTGTGCTC
AGGCCAGCATCGGTTCTCGTGGGGGGAGAAGAGCGGTGGGAATGTGGCTCACCTTCGGGTGAGTGTTATA
GCCCGCCGCACGATGCCCCCGTGGGGACCGAGGTTCGCGCTTCC----
GCATGGATGCTGGCGTAATGGTCACTAGCGACCCGTCTTGAAACACGGACCAAGGAGTCGTCCATTAGAG
CGAGCGTTTGGGTGTCAAACCCGCACGCGTAATGAAAGTGAAAT-----
TAGGTGAGAGCTTCGGCGCATCATCGACCGATCCTGATGTTCTCGGATGGATTTGAGTAAGAGTTTTAAC
GGACGGACCCGAAAGACAGTGAACTATGCTTGTATAGGGTGAAGCCAGAGGAAACTCTGGTGGAGGCTCG
CAGCGGTTCTGACGTGCAAATCGATCGTCAAATATGAGCATGGGGGCGAAAGACTAATCGAAC-------
--TCTCCGTTGGTGAACCAGCGGAGGGATCATTGCTGGAA-GCGTCGCAAGACGCGCCCAG---
ATTACCCCTATGTGAACTTATA----TTATCGTTGCCTCGGTCAAGGCTGGGTCTCCCTG-

AGATCCCCTCTCTGACATCCCCTCCTCCCTCGGGAGGGGAGGACGGACGAAGAGGAGCCGCCGGCCGTTG
GCCC-----TTTACACTCTTG-TCTCCATCTGG---ATTTCTTCTGAGTATAAACAAAC---
AAATGAATCAAAACTTTCAACAACGGATCTCTTGGTTCTGGCATCGATGAAGAACGCAGCGAAATGCGAT
AAGTAATGTGAATTGCAGAATTCAGTGAATCATCGAATCTTTGAACGCACATTGCGCCCGCTGGTATTCC
AGCGGGCATGCCTGTTCGAGCGTCATTTCAACCCTC----AAGCCCAG----------------------
---CTTGGTGTTGGGACATT--GCCATCTCG---TCCATCTTCTGGATGCGCGA---------
GGGCAAGTCCTCAAATTGAGTGGCGAGCTCGCCAGGA---CTCCGAGCGTAGTAGTTT--------
TACCACCTCGCTT--TGGACGGACTGGCGGTGTCTCGGCCGTAAAA--
CCCCCCAATTTATTACCAAAGGTTGACCTCGGATCAGGTAGGAATACCCGCTGAACTTAA----------
----

CCTTCTGGTGTGTACACACAGCTTCACGGCCGCACCGCGTCGATCGGGCACGGCGGTGTTTTGTTATCGT
GACATTGTTACTGACCTCAATTGCAGGCAAACCATCTCTGGCGAGCACGGCCTCGACAGCAATGGTGTGT
ACGTG-------TTAACCCTCCATACCTTCTTGACACTTGTCT--GC--CTTGGAGACTGACCT-
CGCCGCAGTTACAACGGCACCTCCGAGCTGCAGCTCGAGCGCATGAACGTCTACTTCAACGAGGTATGTC
GGTTGGCATTCACGAACCCCACCCCCCTCCCCGCCGCTTTTCGCGTGACAGGTGATCTCGACTGCTTGAT
TAGTATGTCTGACGCGCT------
GTTGTACAGGCCTCCGGCAACAAGTATGTTCCTCGCGCTGTTCTTGTCGACCTCGAGCCCGGTACCATGG
ACGCTGTCCGCGCTGGTCCTTTTGGCCAGCTGTTCCGCCCCGACAACTTCGTCTTCGGCCAGTCCGGTGC
CGGCAACAACTGGGCCAAGGG
>Lasmenia_sp__LMS_2011b_CBS_124122
CGAGTGAAGCGGCAACAGCTCAAATTTGAAATCTGGCCTCGCGGCCCGAGTTGTAATTTGCAGAGGATGC
TTCTGGCGCGGTGCCTTCCGAGTTCCCTGGAACGGGACGCCACAGAGGGTGAGAGCCCCGTCTGGTGGGA
CACCAAGCCTGTGTGAAGCTCCTTCGACGAGTCGAGTAGTTTGGGAATGCTGCTCTAAATGGGAGGTAAA
TCTCTTCTAAAGCTAAATACCGGCCAGAGACCGATAGCGCACAAGTAGAGTGATCGAAAGATGAAAAGCA
CCTTGAAAAGGGGGTTAAACAGTACGTGAAATTGTTGAAAGGGAAGCGCTTGTGACCAGACGTGCGCCCG
GCGGCTCATCCAGCGTTCGCGCTGGTGCACTCCGCCGG-
GCGCAGGCCAGCATCGGTTTCGGCGGGGGGAGAAGGACGGCAGGAATGTGGCTCCCCTCCGGGGGAGTGT

TATAGCCTGCCGTACGATGCCCCCGCGGGGACCGAGGACCGCGCTGTCAAAGGCAAGGATGCTGGCGTAA
TGGTTACTAGCGACCCGTCTTGAAACACGGACCAAGGAGTCGTCCATTGGAGCGAGCGTTTGGGTGTCAA
ACCCGCACGCGCAATGAAAGTGAAATTTCTATAGGTGAGAGCCTCGGCGCATCATCGACCGATCCTGATG
TTCTCGGATGGATTTGAGTAAGAGTTTCAACGGACGGACCCGAAAGACAGTGAACTATGCTTGTATAGGG
TGAAGCCAGAGGAAACTCTGGTGGAGGCTCGCAGCGGTTCTGACGTGCAAATCGATCGTCAAATATGAGC
ATGGGGGCGAAAGACTAATCGAACTGTCTAGTA---------------------
AGGGATCATTGCTGGAA-GCGCCGCCAGGCGCGCCCTGCACCTTTACCCGATGTGAACCTATACC---
CACCGTTGCCTCGGTGC-GGCCGGGTGCCCTCGCGCACCCCCCAACCTGCCCCTTCCCCCTCCCCG-
GGGGCGGGAGCGGCGAGGGAGC--CGGCCCGCCGTTGGCCCCAACTCCAAACCTCTTG-
TTTGTTCCAAACCTGTACCTTCTGAGTT-
GATTTTACACAAAATGAATCAAAACTTTCAACAACGGATCTCTTGGTTCTGGCATCGATGAAGAACGCAG
CGAAATGCGATACGTAATGTGAATTGCAGAATTCAGTGAATCATCGAATCTTTGAACGCACATTGCGCCC
GCTGGTATTCCAGCGGGCATGCCTGTTCGAGCGTCATTTCAACCCCTCCGGACACCCCGCGCCCTCGTGG
TGCGTGTGTGTGCTCCGGGTGTTGGGGCACT--GCCG----TGCTTTGTGCTTTGGCACGA---------
----CGGCAGGCCCTCAAATGCAGTGGCGAGCCCGCTGGCACACCTCCGAGCGCAGTAACCT--------
ACACCTCTCGCTT-CTGGACGGTCCAGCGGTTGCCCGGCCGTAAAA--CCCCCCCAAC------------
----

>Lasmenia_sp__LMS_2011c_CBS_124123

CGAGTGAAGCGGCAACAGCTCAAATTTGAAATCTGGCCTCGCGGCCCGAGTTGTAATTTGCAGAGGATGC
TTCTGGCGCGGTGCCTTCCGAGTTCCCTGGAACGGGACGCCACAGAGGGTGAGAGCCCCGTCTGGTGGGA
CACCAAGCCTGTGTGAAGCTCCTTCGACGAGTCGAGTAGTTTGGGAATGCTGCTCTAAATGGGAGGTAAA
TCTCTTCTAAAGCTAAATACCGGCCAGAGACCGATAGCGCACAAGTAGAGTGATCGAAAGATGAAAAGCA
CCTTGAAAAGGGGGTTAAACAGTACGTGAAATTGTTGAAAGGGAAGCGCTTGTGACCAGACGTGCGCCCG
GCGGCTCATCCAGCGTTCGCGCTGGTGCACTCCGCCGG-
GCGCAGGCCAGCATCGGTTTCGGCGGGGGGAGAAGGACGGCAGGAATGTGGCTCCCCTCCGGGGGAGTGT
TATAGCCTGCCGTACGATGCCCCCGCGGGGACCGAGGACCGCGCTGTCAAAGGCAAGGATGCTGGCGTAA
TGGTTACTAGCGACCCGTCTTGAAACACGGACCAAGGAGTCGTCCATTGGAGCGAGCGTTTGGGTGTCAA
ACCCGCACGCGCAATGAAAGTGAAATTTCTATAGGTGAGAGCCTCGGCGCATCATCGACCGATCCTGATG
TTCTCGGATGGATTTGAGTAAGAGTTTCAACGGACGGACCCGAAAGACAGTGAACTATGCTTGTATAGGG
TGAAGCCAGAGGAAACTCTGGTGGAGGCTCGCAGCGGTTCTGACGTGCAAATCGATCGTCAAATATGAGC
ATGGGGGCGAAAGACTAATCGAACTGTCTAGTA---------------------
AGGGATCATTGCTGGAA-GCGCCGCCAGGCGCGCCCTGCACCTTTACCCGATGTGAACCTATACC---
CACCGTTGCCTCGGTGC-GGCCGGGTGCCCTCGCGCACCCCCCAACCTGCCCCTTCCCCCTCCCCG-
GGGGCGGGAGCGGCGAGGGAGC--CGGCCCGCCGTTGGCCCCAACTCCAAACCTCTTG-
TTTGTTCCAAACCTGTACCTTCTGAGTT-
GATTTTACACAAAATGAATCAAAACTTTCAACAACGGATCTCTTGGTTCTGGCATCGATGAAGAACGCAG
CGAAATGCGATACGTAATGTGAATTGCAGAATTCAGTGAATCATCGAATCTTTGAACGCACATTGCGCCC
GCTGGTATTCCAGCGGGCATGCCTGTTCGAGCGTCATTTCAACCCCTCCGGACACCCCGCGCCCTCGTGG
TGCGTGTGTGTGCTCCGGGTGTTGGGGCACT--GCCG----TGCTTTGTGCTTTGGCACGA---------
----CGGCAGGCCCTCAAATGCAGTGGCGAGCCCGCTGGCACACCTCCGAGCGCAGTAACCT--------
ACACCTCTCGCTT-CTGGACGGTCCAGCGGTTGCCCGGCCGTAAAA--CCCCCCCAAC------------
----

>Lasmenia_sp__LMS_2011a_CBS_124126
CGAGTGAAGCGGCAACAGCTCAAATTTGAAATCTGGCCTCGCGGCCCGAGTTGTAATTTGCAGAGGATGC
TTCTGGCGCGGTGCCTTCCGAGTTCCCTGGAACGGGACGCCACAGAGGGTGAGAGCCCCGTCTGGTGGGA
CACCAAGCCTGTGTGAAGCTCCTTCGACGAGTCGAGTAGTTTGGGAATGCTGCTCTAAATGGGAGGTAAA
TCTCTTCTAAAGCTAAATACCGGCCAGAGACCGATAGCGCACAAGTAGAGTGATCGAAAGATGAAAAGCA
CCTTGAAAAGGGGGTTAAACAGTACGTGAAATTGTTGAAAGGGAAGCGCTTGTGACCAGACGTGCGCCCG
GCGGCTCATCCAGCGTTCGCGCTGGTGCACTCCGCCGG-
GCGCAGGCCAGCATCGGTTTCGGCGGGGGGAGAAGGACGGCAGGAATGTGGCTCCCCTCCGGGGGAGTGT
TATAGCCTGCCGTACGATGCCCCCGCGGGGACCGAGGACCGCGCTGTCAAAGGCAAGGATGCTGGCGTAA
TGGTTACTAGCGACCCGTCTTGAAACACGGACCAAGGAGTCGTCCATTGGAGCGAGCGTTTGGGTGTCAA
ACCCGCACGCGCAATGAAAGTGAAATTTCTATAGGTGAGAGCCTCGGCGCATCATCGACCGATCCTGATG
TTCTCGGATGGATTTGAGTAAGAGTTTCAACGGACGGACCCGAAAGACAGTGAACTATGCTTGTATAGGG
TGAAGCCAGAGGAAACTCTGGTGGAGGCTCGCAGCGGTTCTGACGTGCAAATCGATCGTCAAATATGAGC
ATGGGGGCGAAAGACTAATCGAACTGTCTAGTA---------------------
AGGGATCATTGCTGGAA-GCGCCGCCAGGCGCGCCCTGCACCTTTACCCGATGTGAACCTATACC---
CACCGTTGCCTCGGTGC-GGCCGGGTGCCCTCGCGCACCCCCCAACCTGCCCCTTCCCCCTCCCCG-
GGGGCGGGAGCGGCGAGGGAGC--CGGCCCGCCGTTGGCCCCAACTCCAAACCTCTTG-
TTTGTTCCAAACCTGTACCTTCTGAGTT-
GATTTTACACAAAATGAATCAAAACTTTCAACAACGGATCTCTTGGTTCTGGCATCGATGAAGAACGCAG
CGAAATGCGATACGTAATGTGAATTGCAGAATTCAGTGAATCATCGAATCTTTGAACGCACATTGCGCCC
GCTGGTATTCCAGCGGGCATGCCTGTTCGAGCGTCATTTCAACCCCTCCGGACACCCCGCGCCCTCGTGG
TGCGTGTGTGTGCTCCGGGTGTTGGGGCACT--GCCG----TGCTTTGTGCTTTGGCACGA---------
----CGGCAGGCCCTCAAATGCAGTGGCGAGCCCGCTGGCACACCTCCGAGCGCAGTAACCT--------
ACACCTCTCGCTT-CTGGACGGTCCAGCGGTTGCCCGGCCGTAAAA--CCCCCCCAAC------------
----


```
----------------------------------------------------------------------
----------------------------------------------------------------------
----------------------------------------------------------------------
----------------------------------------------------------------------
----------------------------------------------------------------------
----------------------------------------------------------------------
----------------------------------------------------------------------
----------------------------------------------------------------------
----------------------------------------------------------------------
----------------------------------------------------------------------
----------------------------------------------------------------------
----------------------------------------------------------------------
-----------------
>Lasmenia_sp__LMS_2011e_CBS_124125
CGAGTGAAGCGGCAACAGCTCAAATTTGAAATCTGGCCTCGCGGCCCGAGTTGTAATTTGCAGAGGATGC
TTCTGGCGCGGTGCCTTCCGAGTTCCCTGGAACGGGACGCCACAGAGGGTGAGAGCCCCGTCTGGTGGGA
CACCAAGCCTGTGTGAAGCTCCTTCGACGAGTCGAGTAGTTTGGGAATGCTGCTCTAAATGGGAGGTAAA
TCTCTTCTAAAGCTAAATACCGGCCAGAGACCGATAGCGCACAAGTAGAGTGATCGAAAGATGAAAAGCA
CCTTGAAAAGGGGGTTAAACAGTACGTGAAATTGTTGAAAGGGAAGCGCTTGTGACCAGACGTGCGCCCG
GCGGCTCATCCAGCGTTCGCGCTGGTGCACTCCGCCGG-
GCGCAGGCCAGCATCGGTTTCGGCGGGGGGAGAAGGACGGCAGGAATGTGGCTCCCCTCCGGGGGAGTGT
TATAGCCTGCCGTACGATGCCCCCGCGGGGACCGAGGACCGCGCTGTCAAAGGCAAGGATGCTGGCGTAA
TGGTTACTAGCGACCCGTCTTGAAACACGGACCAAGGAGTCGTCCATTGGAGCGAGCGTTTGGGTGTCAA
ACCCGCACGCGCAATGAAAGTGAAATTTCTATAGGTGAGAGCCTCGGCGCATCATCGACCGATCCTGATG
TTCTCGGATGGATTTGAGTAAGAGTTTCAACGGACGGACCCGAAAGACAGTGAACTATGCTTGTATAGGG
TGAAGCCAGAGGAAACTCTGGTGGAGGCTCGCAGCGGTTCTGACGTGCAAATCGATCGTCAAATATGAGC
ATGGGGGCGAAAGACTAATCGAACTGTCTAGTA---------------------
AGGATCATTGCTGGAA-GCGCCGCCAGGCGCGCCCTGCACCTTTACCCGATGTGAACCTATACC---
CACCGTTGCCTCGGTGC-GGCCGGGTGCCCTCGCGCACCCCCCAACCTGCCCCTTCCCCCTCCCCG-
GGGGCGGGAGCGGCGAGGGAGC--CGGCCCGCCGTTGGCCCCAACTCCAAACCTCTTG-
TTTGTTCCAAACCTGTACCTTCTGAGTT-
GATTTTACACAAAATGAATCAAAACTTTCAACAACGGATCTCTTGGTTCTGGCATCGATGAAGAACGCAG
CGAAATGCGATACGTAATGTGAATTGCAGAATTCAGTGAATCATCGAATCTTTGAACGCACATTGCGCCC
GCTGGTATTCCAGCGGGCATGCCTGTTCGAGCGTCATTTCAACCCCTCCGGACACCCCGCGCCCTCGTGG
TGCGTGTGTGTGCTCCGGGTGTTGGGGCACT--GCCG--------TGCTTTGTGCTTTGGCACGA-----
----CGGCAGGCCCTCAAATGCAGTGGCGAGCCCGCTGGCACACCTCCGAGCGCAGTAACCT--------
ACACCTCTCGCTT-CTGGACGGTCCAGCGGTTGCCCGGCCGTAAAA--CCCCCCCAAC------------
----------------------------------------------------------------------
----------------------------------------------------------------------
----------------------------------------------------------------------
----------------------------------------------------------------------
----------------------------------------------------------------------
----------------------------------------------------------------------
----------------------------------------------------------------------
```


>Pseudoplagiostoma_corymbiae_CBS_132529
CGAGTGAAGCGGCAACAGCTCAAATTTGAAATCTGGCTTCG--
GCCCGAGTTGTAATTTGCAGAGGATGCTTCTGGCGCGGTGCCTTCCGAGTTCCCTGGAACGGGACGCCAT
AGAGGGTGAGAGCCCCGTATGGATGGACACCAAGCCTGTGTGAAGCTCCTTCAACGAGTCGAGTAGTTTG
GGAATGCTGCTCTAAATGGGAGGTAAATCTCTTCTAAAGCTAAATACCGGCCAGAGACCGATAGCGCACA
AGTAGAGTGATCGAAAGATGAAAAGCACCTTGAAAAGGGGGTTAAATAGTACGTGAAATTGTTGAAAGGG
AAGCGCTTGTGACCAGACTTGCGCCAGGCGGCTCATCCGGGGTTCTCCCCGGTGCACTCCGCCTG-
GCTCAGGCCAGCATCGGTTCTTGTGGGGGGACAAGAGCAGTGGGAACGTAGCTC-CCTACGGG-
GAGTGTTATAGCCCACTGTACGATACCCTCGCGGGGACCGAGGACCGCGCTTCG----
GCAAGGATGCTGGCGTAATGGTCACCAGCGACCCGTCTTGAAACACGGACCAAGGAGTCGTCCATTAGAG
CGAGCGTTTGGGTGTCAAACCCGCACGCGTAATGAAAGTGAAAT-----
TAGGTGAGAGCTTCGGCGCATCATCGACCGATCCTGATGTTCTCGGAYGGATTTGAGTAAGAGTTTTAAC
GGACGGACCCGAAAGACAGTGAACTATGCTTGTATAGGGTGAAGCCAGAGGAAACTCTGGTGGAGGCTCG
CAGCGGTTCTGACGTGCAAATCGATCGTCAAATATGAGCATGGGGGCGAAAGACTAATCGAACTGTCTAG
TATCTCCGTTGGTGAACCAGCGGAGGGATCATTGCTGGAA-GCGTCTCACGGCGCACCCAG---AT--
ACCCTTTGTGAACCTGTACC--CTTCTGTTGCCTCGGCGCAGGCTGGGCGCTTCCACACGCCCCC--
ACGCAGGCCTCAACCCCCTGTGCGGAGC-------------------CAGCCGGCCGGTGGCCCACA--
ACCCAACTCTTG-TTTTTACCCA-CCCGTCTC-TCTGAGTA-ACCAAAAC---
AAATGAATCAAAACTTTCAACAACGGATCTCTTGGTTCTGGCATCGATGAAGAACGCAGCGAAATGCGAT
AAGTAATGTGAATTGCAGAATTCAGTGAATCATCGAATCTTTGAACGCACATTGCGCCCGCTGGTATTCC
AGCGGGCATGCCTGTTCGAGCGTCATTTCACCCCTC----AAGCTCCG----------------------
---CTTGGTGTTGGGGCACT--GCCG--------CGCCGCTTCGGCG----------------
GGGCAGGCCCCCAAATCCAGTGGCGAGCTCGCCAGGA---CTCCGAGCGTAGTAGTT---------
ATTCACCTCGCTA--TGGACGGACTGGCGGTGCTT-TGCCGTTAAA--CCCCCCCACTTTCTG--

AAAAGTTGACCTCGGATCAGGTAGGAATACCCGCTGAACTTAA---------------------------
----

>Pseudoplagiostoma_eucalypti_CBS_124807
--G--
GCCCGAGTTGTAATTTGCAGAGGATGCTTCTGGCGCGGTGCCTTCCGAGTTCCCTGGAACGGGACGCCAT
AGAGGGTGAGAGCCCCGTATGGATGGACACCAAGCCTGTGTGAAGCTCCTTCAACGAGTCGAGTAGTTTG
GGAATGCTGCTCTAAATGGGAGGTAAATCTCTTCTAAAGCTAAATACCGGCCAGAGACCGATAGCGCACA
AGTAGAGTGATCGAAAGATGAAAAGCACCTTGAAAAGGGGGTTAAATAGTACGTGAAATTGTTGAAAGGG
AAGCGCTTGTGACCAGACTTGCGCCAGGCGGCTCATCCGGGGTTCTCCCCGGTGCACTCCGCCTG-
GCTCAGGCCAGCATCGGTTCTTGTGGGGGGATAAGACCAGTGGGAACGTAGCTC-CCTACGGG-
GAGTGTTATAGCCCATTGGACGATACCCTCGCGGGGACCGAGGACCGCGCTTCG----
GCAAGGATGCTGGCGTAATGGTCACCAGCGACCCGTCTTGAAACACGGACCAAGGAGTCGTCCATTAGAG
CGAGCGTTTGGGTGTCAAACCCGCACGCGTAATGAAAGTGAAAT-----
TAGGTGAGAGCTTCGGCGCATCATCGACCGATCCTGATGTTCTCGGATGGATTTGAGTAAGAGTTTTAAC
GGACGGACCCGAAAGACAGTGAACTATGCTTGTATAGGGTGAAGCCAGAGGAAACTCTGGTGGAGGCTCG
CAGCGGTTCTGACGTGCA--
--TCTCCGTTGGTGAACCAGCGGAGGGATCATTGCTGGAA-GCGTCTCACGGCGCACCCAG---AT--
ACCCTTTGTGAACCTATACC--CTTCTGTTGCCTCGGCGCAGGCTGGGCGCTTCCACACGCCCCC--
ACGCAGGCCTCAACCCCCTGTGCGGAGC-------------------CAGCCGGCCGGTGGCCCACA--

ACCAAACTCTTG-TTTTTAC--AACCCGTCTC-TCTGAGCA-ACCAAAAC---
AAATGAATCAAAACTTTCAACAACGGATCTCTTGGTTCTGGCATCGATGAAGAACGCAGCGAAATGCGAT
AAGTAATGTGAATTGCAGAATTCAGTGAATCATCGAATCTTTGAACGCACATTGCGCCCGCTGGTATTCC
AGCGGGCATGCCTGTTCGAGCGTCATTTCACCCCTC----AAGCTCCG----------------------
---CTTGGTGTTGGGGCACT--GCCG--------CGCCGCTTCGGCG----------------
AGGCAGGCCCCCAAATGCAGTGGCGGGCTCGCCAGGA---CTCCGAGCGTAGTAGTT---------
ATTCACCTCGCTA--TGGACGGACTGGCGGTGCTT-TGCCGTTAAA--CCCCCCAATTTCT----
GAAAAGTGACCTCGGG--

--------------GAAGGTATGTGTCAAGATCTCCATTT---GTATCAATCCTACATC--
CCGAATTTCAC-TTTGGAACG--AG--TCCAGCATGTC---ATAGCAACCTGGACCGCTGT-
GTTTCTCATTCACTCATTTTTTATCCGCGGGGCGCACATTTTGGATTTTTGGTGCGGGG-
TTTGAGCACCTC-TATCTTT-GATAAGCGTGACCCGCCTCG--CAACACTATCCAGAA-----CTCGCT-
TGCCCCGCCATCACATTTTC-----TA-CCCCCTGCATTCGTTC----CATTGGCAGAAC---------
GTGATACTGACTG-ACTTATCATTATAGCCGCTGAGCTCGGTAAGGGTTCCTTCAAGTA-----------
----

----------CTTTCTGGTGCGTACTCGA--CCTCGT-----CACCACGTCGATCGA----
CGCGACATAAAGATTCCGCGATTGTGATACTGATTCCAATTCTAGGCAGACCATTTCTGGTGAGCACGGC
CTTGATAGCAATGGCGTGTACGTA-------CCTACCATCC-CATCCTCGCG-----
CGTCAGGACCTCCCCAAAACTGACCT-
CAGCTTAGCTACAACGGTACCTCCGAGCTCCAGCTCGAGCGTATGAACGTCTACTTCAACGAGGTATGTC
CACAT-------CGAGA-------TCTCATCAATCTCGAA-
TTGAATACGAATTTACCCTATCGCCGAGCT--TTT-TCTGACGCGCT---
TCCATCATCCAGGCATCCGGCAACAAATATGTTCCTCGCGCCGTCCTTGTCGATCTCGAGCCCGGTACCA
TGGACGCCGTCCGTGCCGGTCCTTTCGGACAGCTGTTCCGTCCCGACAACTTCGTTTTCGGTCAGTCC-
GTGCTGGCA--CACTGGGCCAAGGT
>Pseudoplagiostoma_eucalypti_CPC_14161
--G--
GCCCGAGTTGTAATTTGCAGAGGATGCTTCTGGCGCGGTGCCTTCCGAGTTCCCTGGAACGGGACGCCAT
AGAGGGTGAGAGCCCCGTATGGATGGACACCAAGCCTGTGTGAAGCTCCTTCAACGAGTCGAGTAGTTTG
GGAATGCTGCTCTAAATGGGAGGTAAATCTCTTCTAAAGCTAAATACCGGCCAGAGACCGATAGCGCACA
AGTAGAGTGATCGAAAGATGAAAAGCACCTTGAAAAGGGGGTTAAATAGTACGTGAAATTGTTGAAAGGG
AAGCGCTTGTGACCAGACTTGCGCCAGGCGGCTCATCCGGGGTTCTCCCCGGTGCACTCCGCCTG-
GCTCAGGCCAGCATCGGTTCTTGTGGGGGGATAAGACCAGTGGGAACGTAGCTC-CCTACGGG-

GAGTGTTATAGCCCATTGGACGATACCCTCGCGGGGACCGAGGACCGCGCTTCG----
GCAAGGATGCTGGCGTAATGGTCACCAGCGACCCGTCTTGAAACACGGACCAAGGAGTCGTCCATTAGAG
CGAGCGTTTGGGTGTCAAACCCGCACGCGTAATGAAAGTGAAAT-----
TAGGTGAGAGCTTCGGCGCATCATCGACCGATCCTGATGTTCTCGGATGGATTTGAGTAAGAGTTTTAAC
GGACGGACCCGAAAGACAGTGAACTATGCTTGTATAGGGTGAAGCCAGAGGAAACTCTGGTGGAGGCTCG
CAGCGGTTCTGACGTGCA--
--TCTCCGTTGGTGAACCAGCGGAGGGATCATTGCTGGAA-GCGTCTCACGGCGCACCCAG---AT--
ACCCTTTGTGAACCTATACC--CTTCTGTTGCCTCGGCGCAGGCTGGGCGCTTCCACACGCCCCC--
ACGCAGGCCTCAACCCCCTGTGCGGAGC-------------------CAGCCGGCCGGTGGCCCACA--
ACCAAACTCTTG-TTTTTAC--AACCCGTCTC-TCTGAGCA-ACCAAAAC---
AAATGAATCAAAACTTTCAACAACGGATCTCTTGGTTCTGGCATCGATGAAGAACGCAGCGAAATGCGAT
AAGTAATGTGAATTGCAGAATTCAGTGAATCATCGAATCTTTGAACGCACATTGCGCCCGCTGGTATTCC
AGCGGGCATGCCTGTTCGAGCGTCATTTCACCCCTC----AAGCTCCG----------------------
---CTTGGTGTTGGGGCACT--GCCG--------CGCCGCTTCGGCG----------------
AGGCAGGCCCCCAAATGCAGTGGCGGGCTCGCCAGGA---CTCCGAGCGTAGTAGTT---------
ATTCACCTCGCTA--TGGACGGACTGGCGGTGCTT-TGCCGTTAAA--CCCCCCAATTTCT----
GAAAAGTGACCTCGGG--

--------------GAAGGTATGTGTCAAGATCTCCATTT---GTATCAATCCTACATC--
CCGAATTTCAC-TTTGGAACG--AG--GCCAGCATGTC---ATAGCAACCTGGACCGCTGT-
GTTTCTCATTCACTCATTTTTTATCCGCGGGGCGCACATTTTGGATTTTTGGTGCGGGG-
TTTGAGCACCTC-TATCTTT-GATAAGCGTGACCCGCCTCG--CAACACTATCCAAAA-----CTCGCT-
TGCCCCGCCATCACATTTTC-----TA-CCCCCTGCATTCGTTC----CATTGGCAGAAC---------
GTGATACTGACTG-ACTTATCATTATAGCCGCTGAGCTCGGTAAGGGTTCCTTCAAGTA-----------
----

----------CTTTCTGGTGCGTACTCGA--CCTCGT-----CACCACGTCGATCGA----
CGCGACATAAAGATTCCGCGATTGTGATACTGATTCCAATTCTAGGCAGACCATTTCTGGTGAGCACGGC
CTTGATAGCAATGGCGTGTACGTA-------CCTACCATCC-CATCCTCGCG-----
CGTCAGGACCTCCCCAAAACTGACCT-
CAGCTTAGCTACAACGGTACCTCCGAGCTCCAGCTCGAGCGTATGAACGTCTACTTCAACGAGGTATGTC
CACAT-------CGAGA-------TCTCATCAATCTCGAA-
TTGAATACGAATTTACCCTATCGCCGAGCT--TTT-TCTGACGCGCT---
TCCATCATCCAGGCATCCGGCAACAAATATGTTCCTCGCGCCGTCCTTGTCGATCTCGAGCCCGGTACCA
TGGACGCCGTCCGTGCCGGTCCTTTCGGACAGCTGTTCCGTCCCGACAACTTCGTTTTCGGTCAGTCC-

GTGCTGGCA--CACTGGGCCAAGGT
>Pseudoplagiostoma_oldii_CBS_115722
--G--
GCCCGAGTTGTAATTTGCAGAGGATGCTTCTGGCGCGGTGCCTTCCGAGTTCCCTGGAACGGGACGCCAT
AGAGGGTGAGAGCCCCGTATGGATGGACACCAAGCCTGTGTGAAGCTCCTTCAACGAGTCGAGTAGTTTG
GGAATGCTGCTCTAAATGGGAGGTAAATCTCTTCTAAAGCTAAATACCGGCCAGAGACCGATAGCGCACA
AGTAGAGTGATCGAAAGATGAAAAGCACCTTGAAAAGGGGGTTAAATAGTACGTGAAATTGTTGAAAGGG
AAGCGCTTGTGACCAGACTTGCGCCAGGCGGCTCATCCGGGGTTCTCCCCGGTGCACTCCGCCTG-
GCTCAGGCCAGCATCGGTTCTTGTGGGGGGATAAGACCAGTGGGAACGTAGCTC-CCTACGGG-
GAGTGTTATAGCCCATTGTACGATACCCTCGCGGGGACCGAGGACCGCGCTTCG----
GCAAGGATGCTGGCGTAATGGTCACCAGCGACCCGTCTTGAAACACGGACCAAGGAGTCGTCCATTAGAG
CGAGCGTTTGGGTGTCAAACCCGCACGCGTAATGAAAGTGAAAT-----
TAGGTGAGAGCTTCGGCGCATCATCGACCGATCCTGATGTTCTCGGATGGATTTGAGTAAGAGTTTTAAC
GGACGGACCCGAAAGACAGTGAACTATGCTTGTATAGGGTGAAGCCAGAGGAAACTCTGGTGGAGGCTCG
CAGCGGTTCTGACGTGCA--
--TCTCCGTTGGTGAACCAGCGGAGGGATCATTGCTGGAA-GCGTCTCACGGCGCACCCAG---AT--
ACCCTTTGTGAACCTATACC--CTTCTGTTGCCTCGGCGCAGGCTGGGCGCTTCCACACGCCCCC--
ACGCAGGCCT-AACCCCCTGTGCGGAGC-------------------CAGCCGGCCGGTGGCCCACA--
ACCAAACTCTTG-TTTTTAC--AACCCGTCTC-TCTGAGTA-ACCAAAAC---
AAATGAATCAAAACTTTCAACAACGGATCTCTTGGTTCTGGCATCGATGAAGAACGCAGCGAAATGCGAT
AAGTAATGTGAATTGCAGAATTCAGTGAATCATCGAATCTTTGAACGCACATTGCGCCCGCTGGTATTCC
AGCGGGCATGCCTGTTCGAGCGTCATTTCACCCCTC----AAGCTCCG----------------------
---CTTGGTGTTGGGGCACT--GCCG--------CGCCGCTTCGGCG----------------
AGGCAGGCCCCCAAATGCAGTGGCGGGCTCGCCAGGA---CTCCGAGCGTAGTAGTT---------
ATTCACCTCGCTA--TGGACGGACTGGCGGTGCTT-TGCCGTTAAA--CCCCCCAATTTCT----
GAAAAGTGACCTCGGG--

--------------GAAGGTATGTGTCAAGATCTCCATTT---GTATCAATCCTACATC--
CCGAATTTCACTTTTGGAACG--AG--GTCAGCATGTC---ATAGTATCCTGGACCGCTGT-
GTTTCTCATTCACTCATTTTTTATCCGCGGGGCGCACATTTTGGATTTTTGGTGCGGGG-
TTTGAGCACCTC-TATCTTT-GATAAGCGTGACCCGCCTCG--CAACACTATCCAAAA-----CTCGCT-
TGCCCCGCCATCACATTTTC-----TA-CCCCCTGCATTCGTTC----CATTGGCAGAAC---------
GTGATACTGACTG-ACTTATCATTATAGCCGCTGAGCTCGGTAAGGGTTCCTTCAAGTA-----------
----

----------CTTTCTGGTGCGTACTCGA--CCTCGT-----CACCACATCGATCGA----
CGCGACATAACGATTCCGCGACTGTGATACTGATTCCAATTCTAGGCAGACCATTTCTGGCGAGCACGGC
CTTGATAGCAATGGCGTGTACGTA-------CCTATCATCC-CATCCTCGCG-----
CGTCAGGACCTCCCCAAAACTGACCT-
CAGCTTAGCTACAACGGTACCTCCGAGCTCCAGCTCGAGCGTATGAACGTCTACTTCAACGAGGTATGTC
CACAT-------CAAGA-------TCTCGTCAATCTCGAA-
TTCAATACGAATTTACCCTATCGCCGAGCT--TGC-TCTGACGCGCT---
TCCATCATTCAGGCATCCGGCAACAAATATGTTCCTCGCGCCGTCCTTGTCGATCTCGAGCCCGGTACCA
TGGACGCCGTCCGTGCCGGTCCTTTCGGACAGCTGTTCCGTCCCGACAACTTCGTTTTCGGTCAGTCC-
GTGCTGGCA--CACTGGGCCAAGGT
>Pseudoplagiostoma_oldii_CBS_124808
--G--
GCCCGAGTTGTAATTTGCAGAGGATGCTTCTGGCGCGGTGCCTTCCGAGTTCCCTGGAACGGGACGCCAT
AGAGGGTGAGAGCCCCGTATGGATGGACACCAAGCCTGTGTGAAGCTCCTTCAACGAGTCGAGTAGTTTG
GGAATGCTGCTCTAAATGGGAGGTAAATCTCTTCTAAAGCTAAATACCGGCCAGAGACCGATAGCGCACA
AGTAGAGTGATCGAAAGATGAAAAGCACCTTGAAAAGGGGGTTAAATAGTACGTGAAATTGTTGAAAGGG
AAGCGCTTGTGACCAGACTTGCGCCAGGCGGCTCATCCGGGGTTCTCCCCGGTGCACTCCGCCTG-
GCTCAGGCCAGCATCGGTTCTTGTGGGGGGATAAGACCAGTGGGAACGTAGCTC-CCTACGGG-
GAGTGTTATAGCCCATTGTACGATACCCTCGCGGGGACCGAGGACCGCGCTTCG----
GCAAGGATGCTGGCGTAATGGTCACCAGCGACCCGTCTTGAAACACGGACCAAGGAGTCGTCCATTAGAG
CGAGCGTTTGGGTGTCAAACCCGCACGCGTAATGAAAGTGAAAT-----
TAGGTGAGAGCTTCGGCGCATCATCGACCGATCCTGATGTTCTCGGATGGATTTGAGTAAGAGTTTTAAC
GGACGGACCCGAAAGACAGTGAACTATGCTTGTATAGGGTGAAGCCAGAGGAAACTCTGGTGGAGGCTCG
CAGCGGTTCTGACGTGCA--
--TCTCCGTTGGTGAACCAGCGGAGGGATCATTGCTGGAA-GCGTCTCACGGCGCACCCAG---AT--
ACCCTTTGTGAACCTATACC--CTTCTGTTGCCTCGGCGCAGGCTGGGCGCTTCCACACGCCCCC--
ACGCAGGCCT-AACCCCCTGTGCGGAGC-------------------CAGCCGGCCGGTGGCCCACA--
ACCAAACTCTTG-TTTTTAC--AACCCGTCTC-TCTGAGTA-ACCAAAAC---
AAATGAATCAAAACTTTCAACAACGGATCTCTTGGTTCTGGCATCGATGAAGAACGCAGCGAAATGCGAT
AAGTAATGTGAATTGCAGAATTCAGTGAATCATCGAATCTTTGAACGCACATTGCGCCCGCTGGTATTCC
AGCGGGCATGCCTGTTCGAGCGTCATTTCACCCCTC----AAGCTCCG----------------------
---CTTGGTGTTGGGGCACT--GCCG--------CGCCGCTTCGGCG----------------
AGGCAGGCCCCCAAATGCAGTGGCGGGCTCGCCAGGA---CTCCGAGCGTAGTAGTT---------
ATTCACCTCGCTA--TGGACGGACTGGCGGTGCTT-TGCCGTTAAA--CCCCCCAATTTCT----
GAAAAGTGACCTCGGG--

--------------GAAGGTATGTGTCAAGATCTCCATTT---GTATCAATCCTACATC--
CCGAATTTCACTTTTGGAACG--AG--GTCAGCATGTC---ATAGTATCCTGGACCGCTGT-
GTTTCTCATTCACTCATTTTTTATCCGCGGGGCGCACATTTTGGATTTTTGGTGCGGGG-
TTTGAGCACCTC-TATCTTT-GATAAGCGTGACCCGCCTCG--CAACACTATCCAAAA-----CTCGCT-
TGCCCCGCCATCACATTTTC-----TA-CCCCCTGCATTCGTTC----CATTGGCAGAAC---------
GTGATACTGACTG-ACTTATCATTATAGCCGCTGAGCTCGGTAAGGGTTCCTTCAAGTA-----------
----

----------CTTTCTGGTGCGTACTCGA--CCTCGT-----CACCACATCGATCGA----
CGCGACATAACGATTCCGCGACTGTGATACTGATTCCAATTCTAGGCAGACCATTTCTGGCGAGCACGGC
CTTGATAGCAATGGCGTGTACGTA-------CCTATCATCC-CATCCTCGCG-----
CGTCAGGACCTCCCCAAAACTGACCT-
CAGCTTAGCTACAACGGTACCTCCGAGCTCCAGCTCGAGCGTATGAACGTCTACTTCAACGAGGTATGTC
CACAT-------CAAGA-------TCTCGTCAATCTCGAA-
TTCAATACGAATTTACCCTATCGCCGAGCT--TGC-TCTGACGCGCT---
TCCATCATTCAGGCATCCGGCAACAAATATGTTCCTCGCGCCGTCCTTGTCGATCTCGAGCCCGGTACCA
TGGACGCCGTCCGTGCCGGTCCTTTCGGACAGCTGTTCCGTCCCGACAACTTCGTTTTCGGTCAGTCC-
GTGCTGGCA--CACTGGGCCAAGGT
>Pseudoplagiostoma_variabile_CBS_113067
--G--
GCCCGAGTTGTAATTTGCAGAGGATGCTTCTGGCGCGGTGCCTTCCGAGTTCCCTGGAACGGGACGCCAT
AGAGGGTGAGAGCCCCGTATGGATGGACACCAAGCCTGTGTGAAGCTCCTTCAACGAGTCGAGTAGTTTG
GGAATGCTGCTCTAAATGGGAGGTAAATCTCTTCTAAAGCTAAATACCGGCCAGAGACCGATAGCGCACA
AGTAGAGTGATCGAAAGATGAAAAGCACCTTGAAAAGGGGGTTAAATAGTACGTGAAATTGTTGAAAGGG
AAGCGCTTGTGACCAGACTTGCGCCAGGCGGCTCATCCGGGGTTCTCCCCGGTGCACTCCGCCTG-
GCTCAGGCCAGCATCGGTTCTTGTGGGGGGATAAGACCAGTGGGAACGTAGCTC-CCTACGGG-
GAGTGTTATAGCCCATTGTACGATACCCTCGCGGGGACCGAGGACCGCGCTTCG----
GCAAGGATGCTGGCGTAATGGTCACCAGCGACCCGTCTTGAAACACGGACCAAGGAGTCGTCCATTAGAG
CGAGCGTTTGGGTGTCAAACCCGCACGCGTAATGAAAGTGAAAT-----
TAGGTGAGAGCTTCGGCGCATCATCGACCGATCCTGATGTTCTCGGATGGATTTGAGTAAGAGTTTTAAC
GGACGGACCCGAAAGACAGTGAACTATGCTTGTATAGGGTGAAGCCAGAGGAAACTCTGGTGGAGGCTCG
CAGCGGTTCTGACGTGCA--
--TCTCCGTTGGTGAACCAGCGGAGGGATCATTGCTGGAA-GCGTCTCACGGCGCACCCAG---AT--
ACCCTTTGTGAACCTATACC--CTTCTGTTGCCTCGGCGCAGGCTGGGCGCTTCCACACGCCCCC--
ACGCAGGCCTAAACCCCCTGTGCGGAGC-------------------CAGCCGGCCGGTGGCCCACA--
ACCAAACTCTTG-TTTTTAC--CACCCGTCTC-TCTGAGTA-ACCAAAAC---
AAATGAATCAAAACTTTCAACAACGGATCTCTTGGTTCTGGCATCGATGAAGAACGCAGCGAAATGCGAT
AAGTAATGTGAATTGCAGAATTCAGTGAATCATCGAATCTTTGAACGCACATTGCGCCCGCTGGTATTCC
AGCGGGCATGCCTGTTCGAGCGTCATTTCACCCCTC----AAGCTCCG----------------------
---CTTGGTGTTGGGGCACT--GCCG--------CGCCGCTTCGGCG----------------
AGGCAGGCCCCCAAATCCAGTGGCGAGCTCGCCAGGA---CTCCGAGCGTAGTAGTT---------
ATTCACCTCGCTA--TGGACGGACTGGCGGTGCTT-TGCCGTTAAC--CCCCCCAATTTCT----
GAAAAGTGACCTCGGG--

--------------GAAGGTATGTGTCAAGATCTCCATCT---TTATCAATCCTACATC--
CCGAATTTCAT-TTTTGAATG--AGGAGCCAGCATGTC---ATCGCAGCCTGTACCGCTGT-
GCTTCTCATCCATTCA-TTTTTGCCCGCGGGGCGCACATTTTGGATTTTTGGTGCGGGG-
TTTGAGCATATC-TATCTTT-GATAAGCGTGACCCGCCTCG--CAACACCATCCAAAA-----CTCGCT-
TGCCCCGCCATCACACTTTC-----TA-CCCCCTGCATTCGTTA----CATTGGTAGAAC---------
GTGATACTAATTG-ATTTACCATTACAGCCGCTGAGCTTGGTAAGGGTTCCTTCAAGTA-----------
----

----------CTTTCTGGTGCGTACTCGA--CCTCGT-----CACCACATCGATCGA----
CGCGACATAACGATTCCGCGACTGTGATACTGATTCAAATTCTAGGCAGACCATTTCTGGCGAGCACGGC
CTTGACAGCAATGGCGTGTACGTA-------CCTATCATCC-CATCCTCGCG-----
CGTCATGACCTCCCCAAAACTGACCT-
CAGCTTAGCTACAACGGTACCTCCGAGCTCCAGCTCGAGCGTATGAACGTCTACTTCAACGAGGTATGTC
CACAT-------AAAGA-------TCTCACCAATCTCGAA-
TTGAATACGAATTCACCCTATCGCCGAGCT--TTT-TCTGACGCGCT---
TCCATTATCCAGGCATCCGGCAATAAATATGTTCCTCGCGCCGTCCTTGTCGATCTCGAGCCCGGTACCA
TGGACGCCGTCCGTGCCGGTCCTTTCGGACAGCTGTTCCGTCCCGACAACTTCGTTTTCGGTCAGTCC-
GTGCTGGCA--CACTGGGCCAAGGT
>Pseudoplagiostoma_corymbiicola_CBS_145052
CGAGTGAAGCGGCAACAGCTCAAATTTGAAATCTGGCTTCG--
GCCCGAGTTGTAATTTGCAGAGGATGCTTCTGGCGCGGTGCCTTCCGAGTTCCCTGGAACGGGACGCCAT
AGAGGGTGAGAGCCCCGTATGGATGGACACCAAGCCTGTGTGAAGCTCCTTCAACGAGTCGAGTAGTTTG
GGAATGCTGCTCTAAATGGGAGGTAAATCTCTTCTAAAGCTAAATACCGGCCAGAGACCGATAGCGCACA
AGTAGAGTGATCGAAAGATGAAAAGCACCTTGAAAAGGGGGTTAAATAGTACGTGAAATTGTTGAAAGGG
AAGCGCTTGTGACCAGACTTGCGCCAGGCGGCTCATCCGGGGTTCTCCCCGGTGCACTCCGCCTG-
GCTCAGGCCAGCATCGGTTCTTGTGGGGGGATAAGACCAGTGGGAACGTAGCTC-TCTACGGG-
GAGTGTTATAGCCCATTGTACGATACCCTCGCGGGGACCGAGGACCGCGCTTCG----
GCAAGGATGCTGACGTAATGGTCACCAGCGACCCGTCTTGAAACACGGACCAAGGAGTCGTCCATTAGAG
CGAGCGTTTGGGTGTCAAACCCGCACGCGTAATGAAAGTGAAAT-----
TAGGTGAGAGCTTCGGCGCATCATCGACCGATCCTGATGTTCTCGGATGGATTTGAGTAAGAGTTTTAAC
GGACGGACCCGAAAGACAGTGAACTATGCTTGTATAGGGTGAAGCCAGAGGAAACTCTGGTGGAGGCTCG
CAGCGGTTCTGACGTGCAAATCGATCGTCAAATATGAGCATGGGGGCGAAAGACTAATCGAA--------
--TCTCCGTTGGTGAACCAGCGGAGGGATCATTGCTGGAA-GCGTTTCACGGCGCACCCAG---AT--
ACCCTTTGTGAACCTATACC--CTTCTGTTGCCTCGGCGCAGGCTGGGCGCTTCCACACGCCCCC--

ACGCAGGCCTAAACCCCCTGTGCGGAGC------------------CAGCCGGCCGGTGGCCCCCAA-
CCAAAACTCTTGTTTTTAC--AACCCGTCTC-TCTGAGCA-ACCAAAAC---
AAATGAATCAAAACTTTCAACAACGGATCTCTTGGTTCTGGCATCGATGAAGAACGCAGCGAAATGCGAT
AAGTAATGTGAATTGCAGAATTCAGTGAATCATCGAATCTTTGAACGCACATTGCGCCCGCTGGTATTCC
AGCGGGCATGCCTGTTCGAGCGTCATTTCACCCCTC----AAGCTCCG----------------------
---CTTGGTGTTGGGGCACT--GCCG--------CGCCGCTTCGGCG----------------
AGGCAGGCCCCCAAATGCAGTGGCGAGCTCGCCAGGA---CTCCGAGCGTAGTAGTT---------
ACTCACCTCGCTA--TGGACGGACTGGCGGTGCTT-TGCCGTTAAA--CCCCCCAATTTCTG---
AAAAGTTGACCTCGGATCAGGTAGGAATACCCGCTGAACTTAA---------------------------
----

--------------GAAGGTATGTGTCAAGATCTCTATTT---GTATCAATCCTATACC--
ATGAATTTCA--CTTTGAATG--AGGCGCCAGCATGTC---ATCGCAGCCTGTGCCGCTGT-
GCTTCTCATCCACTCATTTTTTGCCCGCGGGGCGCACATTTTGGATTTTTGGTGCGGGG-
TTTGAGCACCTC-TATCTTT-GATAAGCGTGACCCGCCTCA--CAACACCATCCAAAA-----CTCGCT-
TGCCCCGCCATCACACTTTC-----TACCCCCCTGCATTCGTTC----CATTGGTAGACC---------
TTGATACTAATTG-
ATTTACCATTACAGCCGCTGAGCTCGGTAAGGGTTCCTTCAAGTACGCATGGGTCCTTGACAAGCTCAAG
GCCGAGCGTGAGCGTGGTATCACCATCGACATTGCTCTGTGGAAGTTCGAGACTCCCAAGTACTATGTCA
CCGTCATTGGTATGTCTATCATCCGCTTCCTGTCTGCGAT-CCTGGAAAGCTTTTGCTAACGAT------
----
CCCACCTCCCAGACGCTCCCGGTCACCGTGATTTCATCAAGAACATGCTTTCTGGTGCGTACTCGA--
CCTCGT-----CACCACATCGATCGA----
CGCGACATAACGATTCCGCGACTGTGATACTGATTCCAATTCTAGGCAGACCATTTCTGGCGAGCACGGC
CTCGACAGCAATGGCGTGTACGTA-------CCTATCATCC-CATCCTCGTG-----
CGTCATGACCTCCCCAAAACTGACCT-
CAGCTTAGCTACAACGGTACCTCCGAGCTCCAGCTCGAGCGTATGAACGTCTACTTCAACGAGGTATGTC
CACAT-------AAAGA---

TCTCATCAATCTCGA---
--ATTG---------
>Pseudoplagiostoma_mangiferae_KUMCC_18_0179
---G--
GCCCGAGTTGTAATTTGCAGAGGATGCTTCTGGTGCGGTGCCTTCCGAGTTCCCTGGAACGGGACGCCAT
AGAGGGTGAGAGCCCCGTATGGATGGACACCAAGCCTGTGTGAAGCTCCTTCAACGAGTCGAGTAGTTTG
GGAATGCTGCTCAAAATGGGAGGTAAATCTCTTCTAAAGCTAAATACCGGCCAGAGACCGATAGCGCACA
AGTAGAGTGATCGAAAGATGAAAAGCACCTTGAAAAGGGGGTTAAATAGTACGTGAAATTGTTGAAAGGG

AAGCGCTTGTGACCAGACTTGCGCCAGGCGGCTCATCCGGTGTTCTCACCGGTGCACTCCGCCTG-
GCCCAGGCCAGCATCGGTTCTCGCGGGGGGATAAGAACGGCGGGAACGTGGCTC-CTCACGGG-
GAGTGTTATAGCCCACCGTACGATACCTTCGCGGGGACCGAGGTTCGCGCATCT----
GCAAGGATGCTGGCGTAATGGTCACCAGCGACCCGTCTTGAAACACGGACCAAGGAGTCGTCCATTAGAG
CGAGCGTTTGGGTGTCAAACCCGCACGCGTAATGAAAGTGAAAT-----
TAGGTGAGAGCTTCGGCGCATCATCGACCGATCCTGATGTTCTCGGATGGATTTGAGTAAGAGTTTTAAC
GGACGGACCCGAAAGACAGTGAACTATGCTTGTATAGGGTGAAGCCAGAGGAAACTCTGGTGGAGGCTCG
CAGCGGTTCTGACGTGCAAATCGATCGTCAAATATGAGCATGGGGGCGAAAGACTAATCGAA--------
--TCTCCGTTGGTGAACCAGCGGAGGGATCATTGCTGGAACGCGTCTCACGGCGCACCCAG---AT--
ACCCTATGTGAACTCATACCTTTTTCTGTTGCCTCGGCGCAGGCTGGGAGCTTCTGTAAACTCCCCCACG
CGGGCC--AACCCTCCACGCGGAGC-------------------AGGCCCGCCGGCGGCCCCCA--
ACCAAACTCTTTGTTTTAAC----TGTATCTCTTCTGAGCA--
ACAACAAAAAAAATGAATCAAAACTTTCAACAACGGATCTCTTGGTTCTGGCATCGATGAAGAACGCAGC
GAAATGCGATAAGTAATGTGAATTGCAGAATTCAGTGAATCATCGAATCTTTGAACGCACATTGCGCCCG
CTGGTATTCCAGCGGGCATGCCTGTTCGAGCGTCATTTCAACCCTC----CAGCCTCG------------
-------------CTGGGTGTTGGGGCACG--GCCG--------CTGCTTTCCAGCGG------------
---AGGCCGGCCCTGAAATACAGTGGCGAGCTCGCTAGGA---CTCCGAGCGTAGTAGTT---------
TTCAACCTCGCTA--TGGACGGCCTAGCGGTGCCC-TGCCGTTAAA--CCCCCCAATTTCTG--------
----

>Pseudoplagiostoma_myracrodruonis_URM_7799
-----GAAGCGGCAACAGCTCAAATTTGAAATCTGGCCTTG--
GCCCGAGTTGTAATTTGCAGAGGATGCTTCTGGCGCGGTGCCTTCCGAGTTCCCTGGAACGGGACGCCAT
AGAGGGTGAGAGCCCCGTATGGTTGGCCACCAAGTCTGTGTGAAGGTCCTTCAACGAGTCGAGTAGTTTG
GGAATGCTGCTCAAAATGGGAGGTAAATCTCTTCTAAAGCTAAATACCGGCCAGAGACCGATAGCGCACA
AGTAGAGTGATCGAAAGATGAAAAGCACCTTGAAAAGGGGGTTAAATAGTACGTGAAATTGTTGAAAGGG
AAGCGCTTGTGACCAGACTTGCGCCAGGCGGCTCATCCGGCGTTCTCGCCGGTGCACTCCGCCTG-
GCCCAGGCCAGCATCGGTTTTCGCGGGGGGATAAGAACGGTGGGAACGTGGCTC-CTCACGGG-
GAGTGTTATAGCCCGCCGTACGATACCCTCGCGGGGACCGAGGTTCGCGCATCT----
GCAAGGATGCTGGCGTAATGGTCACCAGCGACCCGTCTTGAAACACGGACCAAGGAGTCGTCCATTAGAG
CGAGCGTTTGGGTGTTAAACCCGCACGCGTAATGAAAGTGAAAT-----
TAGGTGAGAGCTTCGGCGCATCATCGACCGATCCTGATGTTCTCGGATGGATTTGAGTAAGAGTTTTAAC
GGACGGACCCGAAAGACAGTGAACTATGCTTGAATAGGGTGAAGCCAGAGGAAACTCTGGTGGAGGCTCG
CAGCGGTTCTGACGTGCAAATCGATCGTCAAA--------------------------------------
--TCTCCGTTGGTGAACCAGCGGAGGGATCATTGCTGGAA-GCGTCTCACGACGCACCCAG---AC--
ACCCTTTGTGAACATATACC--
ACTCTGTTGCCTCGGCGCGTGCTGGGGGCGCTGCAATGTGCCCTCCCGTCTCCCTG---
CCGCTCTGCGGTGCGGGGGCGGCG---------CGGCCCGCCGGTGGCCC-----ACTTAACTTA---
TTTTGAC--AACCAGTCTCTTCTGAGTA-ATCATTAT---
CAATGAATCAAAACTTTCAACAACGGATCTCTTGGTTCTGGCATCGATGAAGAACGCAGCGAAATGCGAT
AAGTAATGTGAATTGCAGAATTCAGTGAATCATCGAATCTTTGAACGCACATTGCGCCCGCTGGTATTCC
GGCGGGCATGCCTGTTCGAGCGTCATTTCAACCCTC----AAGCCCAG----------------------
---CTTGGTGTTGGGGCACT--GCTG---------CCTGCATTCGCAGG--------------
GGGCAGGCTCTGAAATTTAGTGGCGAGCTCGCCAGGA---CTCCGAGCGTAGTAGTC---------
AATCACCTCGCTT--AGGAAG---

GGGTCACCGGCTGAGCCTATCAAAGATTTCATGGTGCA--------------
ACGGAATATGGAAGTGCTAGAG-------GAGTATGAACCC---------GGCAGCAGC-----------
-------------CCCGATGCAACCAAGATTTTCATTAACGGCACCTGGGTGGGCGTGCATAACGA----
---------GCCT--GCTCATCTTGTCAGCCT--
GGTCCAAGAGCTTCGGCGGAGATGCATTATCTCCCACGAAGTTTCTCTTGTTCGTGATATCCGTGACCGC
GAGTTCAAGATCTTTTCCGATGCGGGACGTGTGATGCGACCATTGTTTGTAGTGAATCAACAAGATAATC
CCGACACCGGGCTTGCGCAGGGTACATTGGCTTTGACCAAAGAACACATCAGCCGCCTCGAGCAGGACGC
CAATCTCGACAAGGACGATGAAGAATACTTTGGCTGGGACGGACTCCAGAACAATGGTGTTATTGAGTAC
CTCGATGCCGAAGAAGAGGAGAGTGCCATGATTTGCATGAGTCCCGAA--------
GACCTAGAAGACTTCCGTCAGCGAAAAGCACGTCCCAAAGACTACCAAGAAGAGGAAGTTTTGGATGATG
-GTAG----------------
GAGCCCGAACGCTCGTGTGGAGGGTATGTACTGATCTCTTCCAAAAAAATATCCATCACG------
AAAGATATCACACTC--------AGCTGCGACATTGGC---GTCAAGGCTGGGT--------
AACACTCAAATCCGGGTTTTCCAC--------CGCACAATTTGGTTTTTCAGTGCGGGG-TTTGG---
CGTCTTATC--A-GATAAACGTGACCCACACTGG-CCAAACGAGTCGGAA-----ATAACC-
AACCCCGTCATTCCACT--------------------------C----AATTCGTA-------------
TCAATGCTAAC--------TCATTACAGCCGCTGAGCTCGGTAAGGGTTCCTTCAAGTA-----------

----

CTATATCTGGCGAGCATGGCCTCGACAGCAATGGCGTGTACGTAACCTGTTCCTGCTGTTC--
GCTATCGCG-----TGTCT--ACAGCTTCG-TACTGACCA-
TGATACAGCTACAACGGCACATCCGAGCTCCAGCTCGAGCGCATGAACGTCTACTTCAATGAGGTATGTT
AACAT-------AGAAC-------CCCAGCCAACGACGGT-
GTAGGCGTCAATTGTGAGTTGAATGAAGCT--
TGGTCTTAACGTGTTCTTTTTGTGCAATAGGCATCCGGCAACAAGTACGTCCCTCGCGCTGTCCTCGTCG
ATCTCGAGCCCGGTACCATGGACGCCGTCCGCGCCGGTCCTTTCGGCCAGCTATTCCGTCCCGACAACTT
CGTCTTTGGCCAGTCTGGTGCTGGAAACAACTGGGCCAAGGG
>Pseudoplagiostoma_myracrodruonis_URM_8123
-----GAAGCGGCAACAGCTCAAATTTGAAATCTGGCCTTG--
GCCCGAGTTGTAATTTGCAGAGGATGCTTCTGGCGCGGTGCCTTCCGAGTTCCCTGGAACGGGACGCCAT
AGAGGGTGAGAGCCCCGTATGGTTGGCCACCAAGTCTGTGTGAAGGTCCTTCAACGAGTCGAGTAGTTTG
GGAATGCTGCTCAAAATGGGAGGTAAATCTCTTCTAAAGCTAAATACCGGCCAGAGACCGATAGCGCACA
AGTAGAGTGATCGAAAGATGAAAAGCACCTTGAAAAGGGGGTTAAATAGTACGTGAAATTGTTGAAAGGG
AAGCGCTTGTGACCAGACTTGCGCCAGGCGGCTCATCCGGCGTTCTCGCCGGTGCACTCCGCCTG-
GCCCAGGCCAGCATCGGTTTTCGCGGGGGGATAAGAACGGTGGGAACGTGGCTC-CTCACGGG-
GAGTGTTATAGCCCGCCGTACGATACCCTCGCGGGGACCGAGGTTCGCGCATCT----
GCAAGGATGCTGGCGTAATGGTCACCAGCGACCCGTCTTGAAACACGGACCAAGGAGTCGTCCATTAGAG
CGAGCGTTTGGGTGTTAAACCCGCACGCGTAATGAAAGTGAAAT-----
TAGGTGAGAGCTTCGGCGCATCATCGACCGATCCTGATGTTCTCGGATGGATTTGAGTAAGAGTTTTAAC
GGACGGACCCGAAAGACAGTGAACTATGCTTGAATAGGGTGAAGCCAGAGGAAACTCTGGTGGAGGCTCG
CAGCGGTTCTGACGTGCAAATCGATCGTCAAA--------------------------------------
--TCTCCGTTGGTGAACCAGCGGAGGGATCATTGCTGGAA-GCGTCTCACGACGCACCCAG---AC--
ACCCTTTGTGAACATATACC--
ACTCTGTTGCCTCGGCGCGTGCTGGGGGCGCTGCAATGTGCCCTCCCGTCTCCCTG---
CCGCTCTGCGGTGCGGGGGCGGCG---------CGGCCCGCCGGTGGCCC-----ACTTAACTNTA--
TTTTGAC--AACCAGTCTCTTCTGAGTA-ATCATTAT---
CAATGAATCAAAACTTTCAACAACGGATCTCTTGGTTCTGGCATCGATGAAGAACGCAGCGAAATGCGAT
AAGTAATGTGAATTGCAGAATTCAGTGAATCATCGAATCTTTGAACGCACATTGCGCCCGCTGGTATTCC
GGCGGGCATGCCTGTTCGAGCGTCATTTCAACCCTC----AAGCCCAG----------------------
---CTTGGTGTTGGGGCACT--GCTG---------CCTGCATTCGCAGG--------------
GGGCAGGCTCTGAAATTTAGTGGCGAGCTCGCCAGGA---CTCCGAGCGTAGTAGTC---------
AATCACCTCGCTT--AGGAAG---

GGGTCACCGGCTGAGCCTATCAAAGATTTCATGGTGCA--------------
ACGGAATATGGAAGTGCTAGAG-------GAGTATGAACCC---------GGCAGCAGC-----------
-------------CCCGATGCAACCAAGATTTCATTAACGGCACCTGGGTGGGCGTGCATAACGA----
---------GCCT--GCTCATCTTGTCAGCCT--
GGTCCAAGAGCTTCGGCGGAGATGCATTATCTCCCACGAAGTTTCTCTTGTTCGTGATATCCGTGACCGC

GAGTTCAAGATCTTTTCCGATGCGGGACGTGTGATGCGACCATTGTTTGTAGTGAATCAACAAGATAATC
CCGACACCGGGCTTGCGCAGGGTACATTGGCTTTGACCAAAGAACACATCAGCCGCCTCGAGCAGGACGC
CAATCTCGACAAGGACGATGAAGAATACTTTGGCTGGGACGGACTCCAGAACAATGGTGTTATTGAGTAC
CTCGATGCCGAAGAAGAGGAGAGTGCCATGATTTGCATGAGTCCCGAA--------
GACCTAGAAGACTTCCGTCAGCGAAAAGCACGTCCCAAAGACTACCAAGAAGAGGAAGTTTTGGATGATG
-GTAG---------------
GAGCCCGAACGCTCGTGTGGAGGGTATGTACTGATCTCTTCCAAAAAAATATCCATCACG------
AAAGATATCACACTC--------AGCTGCGACATTGGC---GTCAAGGCTGGGT--------
AACACTCAAATCCGGGTTTTCCAC--------CGCACAATTTGGTTTTTCAGTGCGGGG-TTTGG---
CGTCTTATC--A-GATAAACGTGACCCACACTG-GCCAAACGAGTCGGAA-----ATAACC-
AACCCCGTCATTCCACT--------------------------C----AATTCGTA-------------
TCAATGCTAAC--------TCATTACAGCCGCTGAGCTCGGTAAGGGTTCCTTCAAGTA-----------
----

CTATATCTGGCGAGCATGGCCTCGACAGCAATGGCGTGTACGTAACCTGTTCCTGCTGTTC--
GCTATCGCG-----TGTCT--ACAGCTTCG-TACTGACCA-
TGATACAGCTACAACGGCACATCCGAGCTCCAGCTCGAGCGCATGAACGTCTACTTCAATGAGGTATGTT
AACAT-------AGAAC-------CCCAGCCAACGACGGT-
GTAGGCGTCAATTGTGAGTTGAATGAAGCT--
TGGTCTTAACGTGTTCTTTTTGTGCAATAGGCATCCGGCAACAAGTACGTCCCTCGCGCTGTCCTCGTCG
ATCTCGAGCCCGGTACCATGGACGCCGTCCGCGCCGGTCCTTTCGGCCAGCTATTCCGTCCCGACAACTT
CGTCTTTGGCCAGTCTGGTGCTGGAAACAACTGGGCCAAGGG
>Pseudoplagiostoma_dipterocarpi_TBRC_1895
CGAGTGAAGCGGCAACAGCTCAAATTTGAAATCTGGCTTCG--
GCCCGAGTTGTAATTTGCAGAGGATGCTTCTGGCGCGGTGCCTTCCGAGTTCCCTGGAACGGGACGCCAT
AGAGGGTGAGAGCCCCGTATGGATGGACACCAAGCCTGTGTGAAGCTCCTTCAACGAGTCGAGTAGTTTG
GGAATGCTGCTCTAAATGGGAGGTAAATCTCTTCTAAAGCTAAATACCGGCCAGAGACCGATAGCGCACA
AGTAGAGTGATCGAAAGATGAAAAGCACCTTGAAAAGGGGGTTAAATAGTACGTGAAATTGTTGAAAGGG
AAGCGCTTGTGACCAGACTTGCGCCAGGCGGCTCATCCGGGGTTCTCCCCGGTGCACTCCGCCTG-
GCTCAGGCCAGCATCGGTTCTTGCGGGGGGATAAGACCAGTGGGAACGTAGCTC-TCTACGGG-
GAGTGTTATAGCCCATTGTACGATACCCTCGCGGGGACCGAGGACCGCGCTTCG----
GCAAGGATGCTGGCGTAATGGTCACCAGCGACCCGTCTTGAAACACGGACCAAGGAGTCGTCCATTAGAG
CGAGCGTTTGGGTGTCAAACCCGCACGCGTAATGAAAGTGAAAT-----
TAGGTGAGAGCTTCGGCGCATCATCGACCGATCCTGATGTTCTCGGATGGATTTGAGTAAGAGTTTTAAC
GGACGGACCCGAAAGACAGTGAACTATGCTTGTATAGGGTGAAGCCAGAGGAAACTCTGGTGGAGGCTCG
CAGCGGTTCTGACGTGCAAATCGATCGTCAAATATGAGCATGGGGGCGAAAGACTAATCGAACTGTCTAG
TA---------------------------CATTGCTGGAA-GCGTCTCACGGCGCACCCAG---AT--
ACCCTTTGTGAACTTATACC--
TATCTGTTGCCTCGGCGCAGGCTGGGAGCTTCTGTAAACTCCCCCACGCGGACTCA---
CCTCCGCGCGGAGC-------------------CGGCCCGCCGGTGGCCC-----TACCAACTCTTG-
TTTTTAT----AGTGTCTCTTCTGAGTT---

ACAAAACAAAAATGAATCAAAACTTTCAACAACGGATCTCTTGGTTCTGGCATCGATGAAGAACGCAGCG
AAATGCGATAAGTAATGTGAATTGCAGAATTCAGTGAATCATCGAATCTTTGAACGCACATTGCGCCCGC
TGGTATTCCAGCGGGCATGCCTGTTCGAGCGTCATTTCAACCCTC----AAGCTCTG-------------
------------CTTGGTGTTGGGGCGCG--GCCG----------CTCCCTCGGGGG-------------
--AGGCCAGCCCTGAAATCTAGTGGCGAGCTCGCTAGGA---CTCCGAGCGTAGTAGTT---------
TTTAACCTCGCTA--TGGACGGACT---

>Pseudoplagiostoma_castaneae_SAUCCmy0162
--G--
GCCCGAGTTGTAATTTGCAAAGGATGTTTCTGGGGCGTTGCCTTCCGAGTTCCCTGGAACGGGACGCCAT
AGAGGGTGACAGCCCCGTATGGTTGGATACCAAGCCTGTGTGAAACTCCTTCGACGAGTCGAGTAGTTTG
AGAATGCTGCTCTAAATGGGAGGTAAATCTCTTCTAAAGCTAAATACCGGCCAGAGACCGATAGCGCACA
AGTAGAGTGATCAAAAGATGAAAAGCCCCTTGAAGGGGGGGTTAAATAGTACGTGAATTTGTTGAAAGGG
AAGCTTTTATGACCAGACTTGTGCCGGGCGTTTCATCCGGTGTTCTCGGCGGTGCACTCCGCCTG-
GCTCAGGCCAGCATCGGTTTCCGTGGGGAGATAAGAGCGGTGGGAACGTGTCTC-TTCACGGG-
GAGTGTTATACCCCGCCGTACGATACCCTCGTGGGGACCGAGGTTCGCGCATCT----
GCAAGGATGCTGATATAATGCTCATCAGCGACCCGTCTTGAAACACGGACCAAGGAGTCGTCCATTAGAG
CGAGCGTTTGGGTGTCAAACCCGCACGCGTAATGAAAGTGAAAT-----

TAGGTGAGAGCTTCGGCGTATCATCGACCGATCCTGATGTTCTCGGATGGATTTGAGTAAGAGTTTTAAC
GGACGGACCCGAAAGACAGTGAACTATGCTTGTATAGGGTGAACCCAGAGGAAACTCTGGTGGAGGCTCG
CAGCTGTTCTGACGTGCA--
--TCTCCCTAGGTGCACCAGCGGAGGGATCATTGCTGGAA-GCGTCTCACGGCGCACCCAG---AA--
ACCCTATGTGAACTTATACC--
ACTTTGTTGCCTCGGCGCGAGCCGGGGGCTTCTATAAGCCCCCCCTTA-----------
CCTCGGTAAGGAGC-------------------AGGCCCGCCGGCGGCCC-----TATAAACTCTTG-
TTTT-----AACGTATCTCTTCTGAGTA----
CAAATTATAAATGAATCAAAACTTTCAACAACGGATCTCTTGGTTCTGGCATCGATGAAGAACGCAGCGA
AATGCGATAAGTAATGTGAATTGCAGAATTCAGTGAATCATCGAATCTTTGAACGCACATTGCGCCCGCT
GGTATTCCAGCGGGCATGCCTGTTCGAGCGTCATTTCAACCCTC----AAGCCCTG--------------
-----------CTTGGTGTTGGGGCGCT--GCCG----------CTCCCTCGGGAG--------------
-AGGCAGGCCCTGAAATCTAGTGGCGAGCTCGCCAGGA---CTCCGAGCGTAGTAGTT---------
TACAACCTCGCTT--AGGAAGGACTGGCGGTGCCC-TGCCGTTAAA--------------------------
----

GGATCACCAGCTGAGCCTATCAAGGACTTCATGGTGCA--------------
GCGGAATATGGAAGTATTGGAG-------GAATACGAGCCT---------GGTAGCAGT-----------
-------------CCTGATGCCACCAAGATTTTCATCAATGGTACTTGGGTTGGTGTACACAATGA----
---------ACCT--GCCCACCTGGTCAGCCT--
CGTGCAAGAGCTTCGACGGAGATGCATTATTTCTCACGAGGTCTCCCTTGTCCGTGATATCCGGGACCGT
GAATTCAAGATTTTCTCGGATGCCGGTCGTGTCATGCGACCTTTGTTCGTGGTTAATCAGCAAGACAATC
CTGAAACTGGAGCGGTACCGGGTACACTGGCTCTGACCAAGGACCATATCAGCCGCCTTGAGCAAGATGC
TGGCCTCGAGAAGGATGATGAAGACTACTTCGGCTGGGATGGGTTGCAAAATAGTGGTGTTATCGAATAC
CTTGATGCCGAGGAAGAAGAGAGTGCAATGATCTGCATGACCCCTGAA--------
GATCTAGAAGATTATCGCCAGAAGAAGGCTCGACCAAAAGGTGCCCAGGAAGACGAGACCGAGGATGACG
-GCAG---------------GAGTCTGAACGCCCGTGTCGAGGGTACGTATTGATCTCCC--------
ATATTCCTCATCTCCC--ATGAATACC-----TAGGAAAACAGTCGCGATTCAATT---ATCGTGA-
CTGCTTCGCCTT--ACCCCCCTCTACACATTTTTT------CAAGCGCACATTTTGG-
TTTTTGGTGCGGGGTTTTGGTCCTTTCTTATCTCA-GATAAACGTGACCCACCTCCG-
CCACCACTACCAGAAGGCCTCTTCCT-TCCCCTTCCTGCATTACCAC-----
CCTACCCCTACATTTGGCCTGATTGTTGGTTGCA----------
ATAATATTAACTTCGTTTCTTATCATAGCTGCTGAGCTCGGTAAGGGTTCCTTCAAG-------------
----

CCATCTCTGGCGAGCACGGCCTCGACAGCAATGGCGTGTACGTATCCGC--CCCGCTACCC--
ACAATCGCC-----TGTCT--GCAGCTTCG-TACTGACCG-
TAACGTAGCTACAACGGCACCTCTGAGCTCCAGCTCGAGCGTATGAATGTCTACTTCAACGAGGTACGTG
GATAT-------
AAAGAAAGAAATCCAATCGATTGTTGATGGATCATGCCAATTACGGATTACACTGGGCT--CCT-
TCTGACACACT-
TTTTTTTTGTCCAGGCTTCCGGCAACAAGTATGTCCCTCGCGCCGTCCTTGTCGATCTCGAGCCCGGTAC

CATGGACGCCGTCCGTGCCGGTCCCTTCGGCCAGCTGTTCCGCCCCGACAACTTCGTCTTCGGCCAGTCC
GGTGCTGGCAACAACTGGGCCAAGGG
>Pseudoplagiostoma_castaneae_SAUCCmy0523
---G--
GCCCGAGTTGTAATTTGCAGAGGATGTTTCTGGCGCGGTGCCTTCCGAGTTCCCTGGAACGGGACGCCAT
AGAGGGTGAGAGCCCCGTATGGTTGGATACCAAGCCTGTGTGAAACTCCTTCGACGAGTCGAGTAGTTTG
GGAATGCTGCTCTAAATGGGAGGTAAATCTCTTCTAAAGCTAAATACCGGCCAGAGACCGATAGCGCACA
AGTAGAGTGATCGAAAGATGAAAAGCACCTTGAAAAGGGGGTTAAATAGTACGTGAAATTGTTGAAAGGG
AAGCGTTTATGACCAGACTTGTGCCAGGCGGTTCATCCGGCGTTCTCGCCGGTGCACTCCGCCTG-
GCTCAGGCCAGCATCGGTTTCCGTGGGGGGATAAGAACGGTGGGAACGTGGCTC-TTCACGGG-
GAGTGTTATAGCCCGCCGTACGATACCCTCGTGGGGACCGAGGTTCGCGCATCT----
GCAAGGATGCTGGCATAATGGTCATCAGCGACCCGTCTTGAAACACGGACCAAGGAGTCGTCCATTAGAG
CGAGCGTTTGGGTGTCAAACCCGCACGCGTAATGAAAGTGAAAT-----
TAGGTGAGAGCTTCGGCGCATCATCGACCGATCCTGATGTTCTCGGATGGATTTGAGTAAGAGTTTTAAC
GGACGGACCCGAAAGACAGTGAACTATGCTTGTATAGGGTGAAGCCAGAGGAAACTCTGGTGGAGGCTCG
CAGCGGTTCTGACGTGCA--
--TCTCCGTTGGTGAACCAGCGGAGGGATCATTGCTGGAA-GCGTCTCACGGCGCACCCAG---AA--
ACCCTATGTGAACTTATACC--
ACTTTGTTGCCTCGGCGCGAGCCGGGGGCTTCTATAAGCCCCCCCTTA-----------
CCTCGGTAAGGAGC-------------------AGGCCCGCCGGCGGCCC-----TATAAACTCTTG-
TTTT-----AACGTATCTCTTCTGAGTA----
CAAATTATAAATGAATCAAAACTTTCAACAACGGATCTCTTGGTTCTGGCATCGATGAAGAACGCAGCGA
AATGCGATAAGTAATGTGAATTGCAGAATTCAGTGAATCATCGAATCTTTGAACGCACATTGCGCCCGCT
GGTATTCCAGCGGGCATGCCTGTTCGAGCGTCATTTCAACCCTC----AAGCCCTG--------------
-----------CTTGGTGTTGGGGCGCT--GCCG----------CTCCCTCGGGAG--------------
-AGGCAGGCCCTGAAATCTAGTGGCGAGCTCGCCAGGA---CTCCGAGCGTAGTAGTT----------
TACAACCTCGCTT--AGGAAGGACTGGCGGTGCCC-TGCCGTTAAA------------------------
----
GGATCACCAGCTGAGCCTATCAAGGACTTTATGGTGCA--------------
GCGGAATATGGAAGTATTGGAG-------GAATACGAGCCT---------GGTAGCAGT-----------
-------------CCTGATGCCACCAAGATTTTCATCAATGGTACTTGGGTTGGCGTGCACAATGA----
----------ACCT--GCCCACCTGGTCAGCCT--
CGTGCAAGAGCTTCGACGGAGATGCATTATTTCTCACGAGGTCTCCCTTGTCCGTGATATCCGGGACCGT
GAATTCAAGATTTTCTCGGATGCCGGTCGTGTCATGCGACCTTTGTTCGTGGTTAATCAGCAAGACAATC
CTGAAACTGGAGCGGTACCGGGTACACTGGCTCTGACCAAGGACCATATCAGCCGCCTTGAGCAAGATGC
TGGCCTCGAGAAGGATGATGAAGACTACTTCGGCTGGGATGGGTTGCAAAATAGTGGTGTTATCGAATAC
CTCGATGCCGAGGAAGAAGAGAGTGCAATGATCTGCATGACCCCTGAA---------
GATCTAGAAGATTATCGCCAGAAGAAGGCTCGACCAAAAGGTGCCCAGGAAGACGAGACCGAGGATGACG
-GAAG---------------GAGTCTGAACGCCCGTGTCGAGGGTACGTATTGATCTCCC--------
ATATTCCTCATCTCCC--ATGAATACC-----TAGGAAAACAGTCGCGATTCAATT---ATCGTGA-
CTGCTTCGCCTT--ACCCCCCTCTACACATTTTTT------CAAGCGCACATTTTGG-
TTTTTGGTGCGGGGTTTTGGTCCTTTCTTATCTCA-GATAAACGTGACCCACCTCCG-
CCACCACTACCAGAAGGCCTCTTCCT-TCCCCTTCCTGCATTACCAC-----
CCTACCCCTACATTTGGCCTGATTGTTGGTTGCA----------

ATAATATTAACTTCGTTTCTTATCATAGCTGCTGAGCTCGGTAAGGGTTCCTTCAAG-------------
----

CCATCTCTGGCGAGCACGGCCTCGACAGCAATGGCGTGTACGTATCC--GCCCCGCTACCC--
ACAATCGCC-----TGTCT--GCAGCTTCG-TACTGACCG-
TAACGTAGCTACAACGGCACCTCTGAGCTCCAGCTCGAGCGTATGAATGTCTACTTCAACGAGGTACGTG
GATAT-------
AAAGAAAGAAATCCAATCGATTGTTGATGGATCATGCCAATTACGGATTACACTGGGCT--CCT-
TCTGACACACT-
TTTTTTTGTCCAGGCTTCCGGCAACAAGTATGTCCCTCGCGCCGTCCTTGTCGATCTCGAGCCCGGTAC
CATGGACGCCGTCCGTGCCGGTCCCTTCGGCCAGCTGTTCCGCCCCGACAACTTCGTCTTCGGCCAGTCC
GGTGCTGGCAACAACTGGGCCAAGGG
>Pseudoplagiostoma_dipterocarpicola_MFLUCC_21_0142
CGAGTGAAGCGGCAACAGCTCAAATTTGAAATCTGGCTTCG--
GCCCGAGTTGTAATTTGCAGAGGATGCTTCTGGCGCGGTGCCTTCCGAGTTCCCTGGAACGGGACGCCAT
AGAGGGTGAGAGCCCCGTATGGATGGACACCAAGCCTGTGTGAAGCTCCTTCGACGAGTCGAGTAGTTTG
GGAATGCTGCTCTAAATGGGAGGTAAATCTCTTCTAAAGCTAAATACCGGCCAGAGACCGATAGCGCACA
AGTAGAGTGATCGAAAGATGAAAAGCACCTTGAAAAGGGGGTTAAATAGTACGTGAAATTGTTGAAAGGG
AAGCGCTTGTGACCAGACTTGCGCCAGGCGGCTCATCCGGTGTTCTCACCGGTGCACTCCGCCTG-
GCTCAGGCCAGCATCGGTTCTCGCGGGGGGATAAGAACGGCGGGAACGTGGCTT-CCTACGGG-
GAGTGTTATAGCCCGCCGTCCGATACCTTCGCGGGGACCGAGGTTCGCGCATCT----
GCAAGGATGCTGGCGTAATGGTCACCAGCGACCCGTCTTGAAACACGGACCAAGGAGTCGTCCATTAGAG
CGAGCGTTTGGGTGTCAAACCCGCACGCGTAATGAAAGTGACAT-----
TAGGTGAGAGCTTCGGCGCATCACCGACCGATCCTGATGTTCTCGGATGGATTTGAGTATGAGTTTTAAC
GGACGGACCCGAAAGACAGTGAACTATGCTTGTATAGGGTGAAGCCAGAGGAAACTCTGGTGGAGGCTCG
CAGCGGTTCTGACGTGCAAATCGATCGTCAAATATGAGCATGGGGGCGAAAGACTAATCGAACTGTCTA-
-----CCGTTGGTGAACCAGCGGAGGGATCATTGCTGGAACGCGTCTCACGGCGCACCCAG---AT--
ACCCTATGTGAACCTATACC--
TCTCTGTTGCCTCGGCGCAGGCTGGGAGCTTCTGTAAACTCCCCCACGCGGACCTCAACCCTCCGCGCGG
AGC-------------------CGGCCCGCCGGTGGCCC-----TACCAACTCTTG-TTTTTAC----
CGTGTCTCCTCTGAGTA-
ACCAAAACAAAAAATGAATCAAAACTTTCAACAACGGATCTCTTGGTTCTGGCATCGATGAAGAACGCAG
CGAAATGCGATAAGTAATGTGAATTGCAGAATTCAGTGAATCATCGAATCTTTGAACGCACATTGCGCCC
GCTGGTATTCCAGCGGGCATGCCTGTCCGAGCGTCATTTCAACCCTC----AAGCTCTG-----------
--------------CTTGGTGTTGGGGCGCG--GCCG----------CTCCCTCCGGGGGG---------
----AGGCCCGCCCTGAAATCGAGCGGCGAGCTCGCTAGGA---CTCCGAGCGTAGTAGTT---------
TTCAACCTCGCTA--TGGACGGACTAGCGGTGCCC-TGCCGTTAAA--CCCCCCAATTTCTG---
AAAAGTTGACCTCGGATCAGGTAGGAATACCCGCTGAACTTAA---------------------------
----

--------------GAAGGTACGTGTTACAAACCCTATC----
ATCTCAATCCAGCACCGTCTAAAATCCAAAC----------TGTCGCAATACTCCC---
ATCGTGGGCAGTCTCACTTT-GCTGCTCTTTCCCTCATTTTTGA--
GGCGGGGCGCACATTTTGGATTTTTGGTGCGGGG-TTTGATCACCTCTTATCTCT-
GATAAGCGTGGCCCACC---

-------------------------------CAACGA---------------------------------
----

----------------------TTCGATCGATCGA----
CGCGACATAATCGTGCTGTCACGCTCGTACTGACCCCGACTACAGGCAGACCATCTCTGGCGAGCACGGT
CTTGACAGCAATGGCGTGTACGTA-------CCTGCCATCC-TAACATCTCG-----TGTCA--
TCTGCTCCG-
TGCTGACCCAAGACTCAGTTACAACGGTACCTCCGAGCTCCAGCTCGAGCGTATGAACGTCTACTTCAAC
GAGGTATGTTATTTG-------CCATT-------
GCCTTTCTGGTGCTTCGCTGAGCGCGGGCTGCCTTGT----------------TCTAACGCGCT------
TCTGCCCAGGCTTCCGGCAACAAGTATGTTCCTCGCGCCGTCCTCGTCGATCTCGAGCCCGGTACCATGG
ACGCCGTCCGTGCTGGTCCTTTTGGTCAGCTTTTCCGTCCCGACAACTTCGTTTTCGGCCAGTCCGGTGC
CGGCAACAACTGGGCCAAGGG
>Pseudoplagiostoma_dipterocarpicola_MFLUCC_21_0114
CGAGTGAAGCGGCAACAGCTCAAATTTGAAATCTGGCTTCG--
GCCCGAGTTGTAATTTGCAGAGGATGCTTCTGGCGCGGTGCCTTCCGAGTTCCCTGGAACGGGACGCCAT
AGAGGGTGAGAGCCCCGTATGGATGGACACCAAGCCTGTGTGAAGCTCCTTCGACGAGTCGAGTAGTTTG
GGAATGCTGCTCTAAATGGGAGGTAAATCTCTTCTAAAGCTAAATACCGGCCAGAGACCGATAGCGCACA
AGTAGAGTGATCGAAAGATGAAAAGCACCTTGAAAAGGGGGTTAAATAGTACGTGAAATTGTTGAAAGGG
AAGCGCTTGTGACCAGACTTGCGCCAGGCGGCTCATCCGGTGTTCTCACCGGTGCACTCCGCCTG-
GCTCAGGCCAGCATCGGTTCTCGCGGGGGGATAAGAACGGCGGGAACGTAGCTT-CCTACGGG-
GAGTGTTATAGCCCGCCGTACGATACCTTCGCGGGGACCGAGGTTCGCGCATCT----
GCAAGGATGCTGGCGTAATGGTCACCAGCGACCCGTCTTGAAACACGGACCAAGGAGTCGTCCATTAGAG
CGAGCGTTTGGGTGTCAAACCCGCACGCGTAATGAAAGTGAAAT-----
TAGGTGAGAGCTTCGGCGCATCATCGACCGATCCTGATGTTCTCGGATGGATTTGAGTAAGAGTTTTAAC
GGACGGACCCGAAAGACAGTGAACTATGCTTGTATAGGGTGAAGCCAGAGGAAACTCTGGTGGAGGCTCG
CAGCGGTTCTGACGTGCAAATCGATCGTCAAATATGAGCATGGGGGCGAAAGACTAATCGAACT------
-------------------GCGGAGGGATCATTGCTGGAACGCGTCTCACGGCGCACCCAG---AT--
ACCCTATGTGAACCTATACC--
TCTCTGTTGCCTCGGCGCAGGCTGGGAGCTTCTGTAAACTCCCCCACGCGGACCTCAACCCTCCGCGCGG
AGC-------------------CGGCCCGCCGGTGGCCC-----TACCAACTCTTG-TTTTTAC----

CGTGTCTCTTCTGAGTA-
ACCAAAACAAAAAATGAATCAAAACTTTCAACAACGGATCTCTTGGTTCTGGCATCGATGAAGAACGCAG
CGAAATGCGATAAGTAATGTGAATTGCAGAATTCAGTGAATCATCGAATCTTTGAACGCACATTGCGCCC
GCTGGTATTCCAGCGGGCATGCCTGTTCGAGCGTCATTTCAACCCTC----AAGCTCTG-----------
--------------CTTGGTGTTGGGGCGCG--GCCG----------CTCCCTCCGGGGGG---------
----AGGCCCGCCCTGAAATCCAGTGGCGAGCTCGCTAGGA---CTCCGAGCGTAGTAGTT---------
TTCAACCTCGCTA--TGGACGGACTAGCGGTGCCC-TGCCGTTAAA--CCCCCCAATTTCTG---
AAAAGTTGACCTCGGATCAGGTAGGAATACCCGCTGAACTTAA---------------------------
----

--------------GAAGGTACGTGTTACAAACCCTATC----
ATCTCAGTCCAGCACCGTCTAAAATCCAAAC----------TGTCGCAATACTTCC---
ATTGTGGGCAGTCTCACTTT-GCTGCTCTTTCCCTCATTTTTGA--
GGCGGGGCGCACATTTTGGATTTTTGGTGCGGGG-TTTGATCACCTCTTATCTCT-
GATAAGCGTGACCCACC---

-------------------------------CAA------------------------------------
----

----CGA--CCTCGT-----CATTCGATCGATCGA----
CGCGACATAATCGTGCTGTTACGCTCGTACTGACCCCGACTACAGGCAGACCATCTCTGGCGAGCACGGT
CTTGACAGCAATGGCGTGTACGTA-------CCTGCCATCC-TAACATCTCG-----TGTCA--
TCTGCTCCG-
TGCTGACCCAAGACTCAGTTACAACGGTACCTCCGAGCTCCAGCTCGAGCGTATGAACGTCTACTTCAAC
GAGGTATGTTATTTG-------CCATT-------
GCCTTTCTGGTGCTTCGCTGAGCGCGGGCTGCCTTGT----------------TCTAACGCGCT------
TCTGCCCAGGCTTCCGGCAACAAGTATGTTCCTCGCGCCGTCCTCGTCGATCTCGAGCCCGGTACCATGG
ACGCCGTCCGTGCTGGTCCTTTTGGTCAGCTTTTCCGTCCCGACAACTTCGTTTTCGGCCAGTCCGGTGC
CGGCAACAACTGGGCCAAGGG
>Nakataea_oryzae_CBS_243_76
CGAGTGAAGCGGCAACAGCTCAAATTTGAAATCTGGCCCTAG-
GCCCGAGTTGTAATTTGCAGAGGATGCTTTTGGTGAAGCGCTTACCGAGTCCCCTGGAACGGGGCGCCAC
AGAGGGTGAGAGCCCCGTATGGTTTGACGCCGAGCCTCTGTAAAGCTCCTTCGACGAGTCGAGTAGTTTG
GGAATGCTGCTCTAAACGGGAGGTAAATTTCTTCTAAAGCTAAATACCGGCCAGAGACCGATAGCGCACA
AGTAGAGTGATCGAAAGATGAAAAGCACTTTGAAAAGAGGGTTAAATAGCACGTGAAATTGTTGAAAGGG
AAGCGCTCGTGACCAGACTTGCGCCGGGCGGATCATCCAGCGTTCTCGCTGGTGCACTCCGCCCG-

GCTCAGGCCAGCATCGATTTTCGCCGGGGGACAAAAGCTTCGGGAACGTGGCTC-TCTTCGGG-
GAGTGTTATAGCCCGTTGCGTAATACCCCGGCGGGGATCGAGGACCGCGCTTCG----
GCAAGGATGCTGGCGTAATGGTCATCAGCGACCCGTCTTGAAACACGGACCGAGGAGTCAAGCATTAGTG
CGAGTGTTTGGGTGTAAAACCCGCACGCGTAATGAAAGTGAACG-----
TAGGTGAGAGCTTCGGCGCATCATCGACCGATCCTGATGTTTTCGGATGGATTTGAGTAGGAGCATTAAC
GCTTGGACCCGAAAGATGGTGAACTATACTTGAATAGGGTGAAGCCAGAGGAAACTCTGGTGGAGGCTCG
CAGCGGTTCTGACGTGCAAATCGATCGTCAAATTTGAGTATAGGGGCGAAAGACTAATCGAACCATCTAG
---------------------------ATCATTACA-----GAGTTGAAAAC---TCCAA----------
CCCCTGTGTACCTTACCT--TTATCGTTGCTTCGGCGGAGG-----------------------------
-----AGTCCCCCCCGGGGGAGGGCG--------------------CCGCCGGAGGT-------
CACAAACTCTGA-ATTT-----ACAGTGTATC-TCTGAGGA--AAAAAAC---AAAT-
AATTAAAACTTTCAACAACGGATCTCTTGGTTCTGGCATCGATGAAGAACGCAGCGAAATGCGATAAGTA
ATGTGAATTGCAGAATTCAGTGAATCATCGAATCTTTGAACGCACATTGCGCCCGCCGGTATTCCGGCGG
GCATGCCTGTTCGAGCGTCATTTCACCACTC----AAGCCCAG-------------------------
CTTGGTGTTGGGGCACCCGGCCG-----CCCAGCGGCTTCGG-----------------------
GGCCCCCAAGTACATCGGCGGACCCGCTGGGG---
CCCTGAGCGCAGTAACTCGCGGTAAAACGCGCCTCGCTCGGTGGATCCCCGGAGGGCATTCCAGCCGCTA
AA--CCCCCTAATTTTCTT----AGGTTGACCTCGGATCAGGTAGGAATACC------------
TGGACGCCATCCGGACACGC----
GACCCCAAGGTGCGCTTCGCCCGCGTCTGGGCAATGTGCAAGGACAAGAAGTTTTGCGAAAACGAAACTA
CGAAGAAGGGTGGCGACAAAGAGGGGTTTGAGTCGTCAAAACCCCAGGTCACCAAGGTGC---
CGCACGGTGGCTGTGGCAGCGCGCACCCCGACATCCGTCAGAAGGCCTTGGCGCTCTTTGCCAGGGTAAA
GGGCGAGAACAACGACGAGGAAG-----GCGGCCAAAAGA------------AGGAGATCAAGGA-----
----CAAGCCTATCTCGGCCG--AGACGGCCCATGCCATCTT-----------------
CAAGAAGATCTCAGATGAGGACCTGT----GGAACATGGGC-CTGAACAAGGACTACGC---
GCGCCCCGAGTGGCTTA------TCGTCACGG-----------
TTCTTCCCGTTCCTCCTCCTCCAGTACGGC--------CTAGTATCTCTATG-----------
GACGGTACAGGCACGGGCATGCGAAACGAGGACGATTTGACATACAAGCTCGGCGACATCA-----
TCCGTGCCAACGGAAACGTGAAGCAGGCGATCCAAGATGGCGCGCCGGCCCACATCTGCCTCGAGTTCGA
GGAGTTGCTGC---

>Pseudoplagiostoma_alsophilae_SAUCC_WZ0451
CGAGTGAAGCGGCAACAGCTCAAATTTGAAATCTGGCTTCG--
GCCCGAGTTGTAATTTGCAGAGGATGCTTCTGGCGCGGTGCCTTCCGAGTTCCCTGGAACGGGACGCCAC
AGAGGGTGAGAGCCCCGTATGGATGGACACCAAGCCTGTGTGAAGCTCCTTCGACGAGTCGAGTAGTTTG
GGAATGCTGCTCTAAATGGGAGGTAAATCTCTTCTAAAGCTAAATACCGGCCAGAGACCGATAGCGCACA
AGTAGAGTGATCGAAAGATGAAAAGCACCTTGAAAAGGGGGTTAAATAGTACGTGAAATTGTTGAAAGGG
AAGCGCTTGTGACCAGACTTGCGCCAGGCGGCTCACCCGGTGTTCTCACCGGTGCACTCCGCCTG-
GCTCAGGCCAGCATCGGTTCTCGCGGGGGGACAAGAACGGTGGGAACGTAGCTC-CTCACGGG-
GAGTGTTATAGCCCGCCGTACGATACCTTCGCGGGGACCGAGGTTCGCGCATCT----
GCTAGGATGCTGGCGTAATGGTCACCAGCGACCCGTCTTGAAACACGGACCAAGGAGTCGTCCATTAGAG
CGAGCGTTTGGGTGTCAAACCCGCACGCGTAATGAAAGTGAAAT-----
TAGGTGAGAGCTTCGGCGCATCATCGACCGATCCTGATGTTCTCGGATGGATTTGAGTAAGAGTTTTAAC
GGACGGACCCGAAAGACAGTGAACTATGCTTGTATAGGGTGAAGCCAGAGGAAACTCTGGTGGAGGCTCG
CAGCGGTTCTGACGTGCAAATCGATCGTCAAATATGAGCATGGGGGCGAAAGACTAATCGAACTGTCTAG
TATCTCCGTTGGTGAACCAGCGGAGGGATCATTGCTGGAA-GCGTCTCACGGCGCACCCAG---AT--
ACCCTTTGTGAACCTATACC--
TCTCTGTTGCCTCGGCGCAGGCTGGGAGCTTCTGTAAACTCCCCCACGCGGACTCG---
CCTCCGCGCGGAGC-------------------AGGCCCGCCGGCGGCCCCAA--
CCCAACCTCTTTGTTTTTAT----AGTGTCTCTTCTGAGTA---
ACAAACAAAAAATGAATCAAAACTTTCAACAACGGATCTCTTGGTTCTGGCATCGATGAAGAACGCAGCG
AAATGCGATAAGTAATGTGAATTGCAGAATTCAGTGAATCATCGAATCTTTGAACGCACATTGCGCCCGC
TGGTATTCCAGCGGGCATGCCTGTTCGAGCGTCATTTCAACCCTC----AAGCTCTG-------------
------------CTTGGTGTTGGGGCACT--
GCTGGCCCTGCCGCCCTCCTCCGGGGGGGACGGACAGGA--
GGGCAGGCCCTGAAATACAGTGGCGGGCTCGCTAGGA---CTCCGAGCGTAGTAGTTT--------
TACCACCTCGCTA--TGGACGGACTGGCGGTGCTC-TGCCGTAAAA--CCCCCCAATTTCTG---
AAAAGTTGACCTCGGATCAGGTAGGAATACCCGCTGAACTTAAGGATCACCGGCTGAGCCCATCAAAGAT
TTCATGGTACA--------------GCGGAACATGGAAGTGCTGGAG-------GAGTATGAACCC----
-----GGCAGCAGT------------------------
CCTGATGCCACCAAAATCTTCATCAACGGTACATGGGTCGGCGTTCATAACGA-------------
ACCG--GCGCACCTGGTCAGTCT--
TGTGCAGGAGCTTCGGCGAAGATGTATCATCTCGCACGAGGTTTCGCTTGTTCGTGATATCCGTGACCGT
GAGTTCAAGATCTTCTCAGACGCCGGGCGCGTGATGCGACCTTTGTTCGTGGTCAATCAACAGGACAACC
CGGAGACCGGAGCAGTGCAAGGCTCCTTGGCCTTGAGCAAGGAACACATCACCCGCCTTGAACAAGATGC
TGGTCTTGACAAGGATGACGATGAGTACTTTGGTTGGGACGGCTTGCAGAACAACGGTGTCATCGAGTAC
CTCGATGCCGAGGAGGAAGAGACTGCAATGATTTGCATGACCCCTGAG--------
GATCTGGAGGATTACCGACAGCGGAAGCTTCGGCCAAAGGACATGCAAGATGAAGAGCTTGAGGACGACG
-GCCG---------------
GAGCTTGAACGCTCGTGTCGAAGGTACGTGACAATATTTCCCTACACAACTCAAATTCAGCCCC--
GCGAAATCCTCACTACAAAGAACAACCGCCGACACCCT---GCCGCGGCCTGTTTGGTCCG-
CCTACCCACCCACCTAATTTTT-TCGGCGGGGCGCACACATTGGA-TTTTGGTGCGGGG-
TTTTATCATCTC-TATCTTT-GATAAGCGTGACCCACCACG--CTGCCCTCCCTGACA---------

CTGCTGCTGTTCATCACACTC-------------------------C----AATTGGCAAGACCG-----
--ACGATGCTAACTC-
AATTGTTACTACAGCCGCTGAACTCGGTAAGGGTTCCTTCAAGTACGCATGGGTCCTTGACAAGCTCAAG
GCCGAGCGTGAGCGTGGTATCACCATCGACATTGCTCTGTGGAAGTTCGAAACTCCTAAGTACTATGTCA
CCGTCATCGGTATGTGCGATCGCCTTTTCTCGTCAGTCTGC-------AATACGAGCTAACCGA------
----
AGTCCAATCCAGACGCTCCCGGTCACCGTGATTTCATCAAGAACATGCTTTCTGGTGCGTACTCGA--
CCTCGC-----CACTTGATCGATTGA----
CGCGACGTAGCGATGTCACGACATTCATACTGATTACAATTCTAGGCAAACCATCTCTGGCGAGCACGGC
CTCGACAGCAATGGCGTGTACGTG-------CCTGCCAACA-TACAACTGCG-----TGTC---
GCCGCTGAAGTACTGACCG-
AAGCTCAGCTACAATGGTACCTCCGAGCTCCAGCTCGAGCGTATGAACGTCTACTTCAACGAGGTATGTT
CGCA----------AGA-------ACTCACCGTCCACGTC-
CCGGGCACGAATCGCCACTATCGCCCAGGC--TCC-TCTAACGCGCT--
CTATTTTGTGTAGGCTTCCGGCAACAAGTATGTTCCTCGCGCCGTCCTCGTCGATCTCGAGCCCGGTACC
ATGGACGCCGTCCGTGCCGGTCCTTTCGGTCAGCTGTTCCGTCCCGACAACTTCGTTTTCGGCCAGTCCG
GTGCTGGCAACAACTG---------
>Pseudoplagiostoma_alsophilae_SAUCC_WZ0152
CGAGTGAAGCGGCAACAGCTCAAATTTGAAATCTGGCTTCG--
GCCCGAGTTGTAATTTGCAGAGGATGCTTCTGGCGCGGTGCCTTCCGAGTTCCCTGGAACGGGACGCCAC
AGAGGGTGAGAGCCCCGTATGGATGGACACCAAGCCTGTGTGAAGCTCCTTCGACGAGTCGAGTAGTTTG
GGAATGCTGCTCTAAATGGGAGGTAAATCTCTTCTAAAGCTAAATACCGGCCAGAGACCGATAGCGCACA
AGTAGAGTGATCGAAAGATGAAAAGCACCTTGAAAAGGGGGTTAAATAGTACGTGAAATTGTTGAAAGGG
AAGCGCTTGTGACCAGACTTGCGCCAGGCGGCTCACCCGGTGTTCTCACCGGTGCACTCCGCCTG-
GCTCAGGCCAGCATCGGTTCTCGCGGGGGGACAAGAACGGTGGGAACGTAGCTC-CTCACGGG-
GAGTGTTATAGCCCGCCGTACGATACCTTCGCGGGGACCGAGGTTCGCGCATCT----
GCTAGGATGCTGGCGTAATGGTCACCAGCGACCCGTCTTGAAACACGGACCAAGGAGTCGTCCATTAGAG
CGAGCGTTTGGGTGTCAAACCCGCACGCGTAATGAAAGTGAAAT-----
TAGGTGAGAGCTTCGGCGCATCATCGACCGATCCTGATGTTCTCGGATGGATTTGAGTAAGAGTTTTAAC
GGACGGACCCGAAAGACAGTGAACTATGCTTGTATAGGGTGAAGCCAGAGGAAACTCTGGTGGAGGCTCG
CAGCGGTTCTGACGTGCAAATCGATCGTCAAATATGAGCATGGGGGCGAAAGACTAATCGAACTGTCTAG
TATCTCCGTTGGTGAACCAGCGGAGGGATCATTGCTGGAA-GCGTCTCACGGCGCACCCAG---AT--
ACCCTTTGTGAACCTATACC--
TCTCTGTTGCCTCGGCGCAGGCTGGGAGCTTCTGTAAACTCCCCCACGCGGACTCG---
CCTCCGCGCGGAGC-------------------AGGCCCGCCGGCGGCCCCAA--
CCCAACCTCTTTGTTTTTAT----AGTGTCTCTTCTGAGTA---
ACAAACAAAAAATGAATCAAAACTTTCAACAACGGATCTCTTGGTTCTGGCATCGATGAAGAACGCAGCG
AAATGCGATAAGTAATGTGAATTGCAGAATTCAGTGAATCATCGAATCTTTGAACGCACATTGCGCCCGC
TGGTATTCCAGCGGGCATGCCTGTTCGAGCGTCATTTCAACCCTC----AAGCTCTG-------------
------------CTTGGTGTTGGGGCACT--
GCTGGCCCTGCCGCCCTCCTCCGGGGGGGACGGACAGGA--
GGGCAGGCCCTGAAATACAGTGGCGGGCTCGCTAGGA---CTCCGAGCGTAGTAGTTT--------
TACCACCTCGCTA--TGGACGGACTGGCGGTGCTC-TGCCGTAAAA--CCCCCCAATTTCTG---
AAAAGTTGACCTCGGATCAGGTAGGAATACCCGCTGAACTTAAGGATCACCGGCTGAGCCCATCAAAGAT

TTCATGGTACA--------------GCGGAACATGGAAGTGCTGGAG-------GAGTATGAACCC----
-----GGCAGCAGT------------------------
CCTGATGCCACCAAAATCTTCATCAACGGTACATGGGTCGGCGTTCATAACGA-------------
ACCG--GCGCACCTGGTCAGTCT--
TGTGCAGGAGCTTCGGCGAAGATGTATCATCTCGCACGAGGTTTCGCTTGTTCGTGATATCCGTGACCGT
GAGTTCAAGATCTTCTCAGACGCCGGGCGCGTGATGCGACCTTTGTTCGTGGTCAATCAACAGGACAACC
CGGAGACCGGAGCAGTGCAAGGCTCCTTGGCCTTGAGCAAGGAACACATCACCCGCCTTGAACAAGATGC
TGGTCTTGACAAGGATGACGATGAGTACTTTGGTTGGGACGGCTTGCAGAACAACGGTGTCATCGAGTAC
CTCGATGCCGAGGAGGAAGAGACTGCAATGATTTGCATGACCCCTGAG--------
GATCTGGAGGATTACCGACAGCGGAAGCTTCGGCCAAAGGACATGCAAGATGAAGAGCTTGAGGACGACG
-GCCG---------------
GAGCTTGAACGCTCGTGTCGAAGGTACGTGACAATATTTCCCTACACAACTCAAATTCAGCCCC--
GCGAAATCCTCACTACAAAGAACAACCGCCGACACCCT---GCCGCGGCCTGTTTGGTCCG-
CCTACCCACCCACCTAATTTTT-TCGGCGGGGCGCACACTTTGGA-TTTTGGTGCGGGG-
TTTGATCATCTC-TATCTTT-GATAAGCGTGACCCACCACG--CTGCCCTCCCTGACA---------
CTGCTGCTGTTCATCACACTC-------------------------C----AATTGGCAAGACCG-----
--ACGATGCTAACTT-
AATTGTTACTACAGCCGCTGAACTCGGTAAGGGTTCCTTCAAGTACGCATGGGTCCTTGACAAGCTCAAG
GCCGAGCGTGAGCGTGGTATCACCATCGACATTGCTCTGTGGAAGTTCGAAACTCCTAAGTACTATGTCA
CCGTCATCGGTATGTGCGATCGCCTTTTCTCGTCAGTCTGC-------AATACGAGCTAACCGA------
----
AGTCCAATCCAGACGCTCCCGGTCACCGTGATTTCATCAAGAACATGCTTTCTGGTGCGTACTCGA--
CCTCGC-----CACTTGATCGATTGA----
CGCGACGTAGCGATGTCACGACATTCATACTGATTACAATTCTAGGCAAACCATCTCTGGCGAGCACGGC
CTCGACAGCAATGGCGTGTACGTG-------CCTGCCAACA-TACAACTGCG-----TGTC---
GCCGCTGAAGTACTGACCG-
AAGCTCAGCTACAATGGTACCTCCGAGCTCCAGCTCGAGCGTATGAACGTCTACTTCAACGAGGTATGTT
CGCA----------AGA-------ACTCACCGTCCACGTC-
CCGGGCACGAATCGCCACTATCGCCCAGGC--TCC-TCTAACGCGCT--
CTATTTTGTGTAGGCTTCCGGCAACAAGTATGTTCCTCGCGCCGTCCTCGTCGATCTCGAGCCCGGTACC
ATGGACGCCGTCCGTGCCGGTCCTTTCGGTCAGCTGTTCCGTCCCGACAACTTCGTTTTCGGCCAGTCCG
GTGCTGGCAACAACTG---------
>Pseudoplagiostoma_machilus_BW0233
CGAGTGAAGCGGCAACAGCTCAAATTTGAAATCTGGCCTCG--
GCCCGAGTTGTAATTTGCAGAGGATGCTTCTGGCGCGGTGCCTTCCGAGTTCCCTGGAACGGGACGCCAC
AGAGGGTGAGAGCCCCGTATGGATGGACACCAAGCCTGTGTGAAGCTCCTTCGACGAGTCGAGTAGTTTG
GGAATGCTGCTCTAAATGGGAGGTAAATCTCTTCTAAAGCTAAATACCGGCCAGAGACCGATAGCGCACA
AGTAGAGTGATCGAAAGATGAAAAGCACCTTGAAAAGGGGGTTAAATAGTACGTGAAATTGTTGAAAGGG
AAGCGCTTGTGACCAGACTTGCGCCAGGCGGCTCATCCGGTGTTCTCACCGGTGCACTCCGCCTG-
GCCCAGGCCAGCATCGGTTCTCGCGGGGGGACAAGAACGGTGGGAACGTAGCTC-CTCACGGG-
GAGTGTTATAGCCCGCCGTACGATACCCTCGCGGGGACCGAGGTTCGCGCATCT----
GCAAGGATGCTGGCGTAATGGTCACCAGCGACCCGTCTTGAAACACGGACCAAGGAGTCGTCCATTAGAG
CGAGCGTTTGGGTGTCAAACCCGCACGCGTAATGAAAGTGAAAT-----
TAGGTGAGAGCTTCGGCGCATCATCGACCGATCCTGATGTTCTCGGATGGATTTGAGTAAGAGTTTTAAC

GGACGGACCCGAAAGACAGTGAACTATGCTTGTATAGGGTGAAGCCAGAGGAAACTCTGGTGGAGGCTCG
CAGCGGTTCTGACGTGCAAATCGATCGTCAAATATGAGCATGGGGGCGAAAGACTAATCGAACTGTCTAG
TATCTCCGTTGGTGAACCAGCGGAGGGATCATTGCTGGAA-GCGTCTCACGGCGCGCCCAG---GT--
ACCCTTTGTGAACTTATACC--
TATCTGTTGCCTCGGCGCAGGCTGGGAGCTTCTGTAAACTCCCCCACGCGGACTCA---
CCTCCGCGCGGAGC-------------------AGGCCCGCCGGCGGCCCC----
CCCCAACTCTTTGTTTTTAT----AGTATCTCTTCTGAGGA-ACCCAAA----
AAATGAATCAAAACTTTCAACAACGGATCTCTTGGTTCTGGCATCGATGAAGAACGCAGCGAAATGCGAT
ACGTAATGTGAATTGCAGAATTCAGTGAATCATCGAATCTTTGAACGCACATTGCGCCCGCTGGTATTCC
AGCGGGCATGCCTGTTCGAGCGTCATTTCAACCCTC----AAGCTCTG----------------------
---CTTGGTGTTGGGGCCCT--
GCCGGCCCTGCCGACCCCCTTCTCCGGGGGGCGGACTAGGAAGGCAGGCCCTGAAATACAGTGGCGGGCT
CGCTAGGA---CTCCGAGCGTAGTAGTTT--------TACCACCTCGCTA--
TGGACGAACTAGCGGTGCCC-TGCCGTAAAAAAACCCCCTATTTCTG---
AAAAGTTGACCTCGGATCAGGTAGGAATACCCGCTGAACTTAA---------------------------
----

--------------GAAGGTATGTGACAATATTTCCGTGT---GTTTCAATACAACCCC--
GCGAGTTTCATACCGCCAACA--CCACGCCGTAACGTT---GTCGCGGCTTATTTGGTCCG-
CCTGCCCATCCACCCATTTTTT--CGGCGGGGCGCACACTTCGAA-TTTTGGTGCGGGG-
TTTGATCACCTC-TATCACT-GATAAGCGTGACCCACAACG--CTGCCCTCCCTTACA------------
------TCCTTCACACTT-------------------------C----AATTGGCAAGACCG-------
ATGACGCTAATAA-
AATTGTTACAACAGCCGCTGAACTCGGTAAGGGTTCCTTCAAGTACGCATGGGTCCTTGACAAGCTCAAG
GCCGAGCGTGAGCGTGGTATCACCATCGATATTGCTCTGTGGAAGTTCGAGACTCCCAAGTACTATGTCA
CCGTCATTGGTATGTTGCAATCGCCTTTCTCCCTGTCATTACTGAAAAAACACGAGCTAACTGC------
----
AGTCGAATCTAGACGCTCCCGGTCACCGTGACTTCATCAAGAACATGCTTTCTGGTGCGTACTCGACCCC
TCAC-----CACTTCACGAATCGA----
AGCGACGTCGCGATGCCACGACATTCAAACTGATCCCCATTCTAGGCAAACCATCTCTGGCGAGCATGGC
CTCGACAGCAATGGCGTGTACGTA-------CTTGTCATCC-TACAACTGCG-----TGTC---
ACAGCTTAGATACTGACCG-
AAGCTCAGCTACAACGGTACCTCGGAGCTCCAGCTCGAGCGTATGAACGTCTACTTCAACGAGGTATGTT
CGCAG-------AGATT-----------
CCCGTCCACGTCTCCGGGCGCGAATTGCTACTATCGCCAAAGC--TCC-TCTAACGCGCA--
CCATTTCGAGTAGGCCTCCGGCAACAAATATGTTCCTCGCGCCGTTCTTGTCGATCTCGAGCCCGGTACC
ATGGACGCCGTGCGCGCCGGTCCTTTCGGTCAGCTGTTCCGTCCCGACAACTTCGTTTTCGGCCAGTCCG

GTGCTGGCAACAACTGGGCCAAGGG
>Pseudoplagiostoma_machilus_BW0221
CGAGTGAAGCGGCAACAGCTCAAATTTGAAATCTGGCCTCG--
GCCCGAGTTGTAATTTGCAGAGGATGCTTCTGGCGCGGTGCCTTCCGAGTTCCCTGGAACGGGACGCCAC
AGAGGGTGAGAGCCCCGTATGGATGGACACCAAGCCTGTGTGAAGCTCCTTCGACGAGTCGAGTAGTTTG
GGAATGCTGCTCTAAATGGGAGGTAAATCTCTTCTAAAGCTAAATACCGGCCAGAGACCGATAGCGCACA
AGTAGAGTGATCGAAAGATGAAAAGCACCTTGAAAAGGGGGTTAAATAGTACGTGAAATTGTTGAAAGGG
AAGCGCTTGTGACCAGACTTGCGCCAGGCGGCTCATCCGGTGTTCTCACCGGTGCACTCCGCCTG-
GCCCAGGCCAGCATCGGTTCTCGCGGGGGGACAAGAACGGTGGGAACGTAGCTC-CTCACGGG-
GAGTGTTATAGCCCGCCGTACGATACCCTCGCGGGGACCGAGGTTCGCGCATCT----
GCAAGGATGCTGGCGTAATGGTCACCAGCGACCCGTCTTGAAACACGGACCAAGGAGTCGTCCATTAGAG
CGAGCGTTTGGGTGTCAAACCCGCACGCGTAATGAAAGTGAAAT-----
TAGGTGAGAGCTTCGGCGCATCATCGACCGATCCTGATGTTCTCGGATGGATTTGAGTAAGAGTTTTAAC
GGACGGACCCGAAAGACAGTGAACTATGCTTGTATAGGGTGAAGCCAGAGGAAACTCTGGTGGAGGCTCG
CAGCGGTTCTGACGTGCAAATCGATCGTCAAATATGAGCATGGGGGCGAAAGACTAATCGAACTGTCTAG
TATCTCCGTTGGTGAACCAGCGGAGGGATCATTGCTGGAA-GCGTCTCACGGCGCGCCCAG---GT--
ACCCTTTGTGAACTTATACC--
TATCTGTTGCCTCGGCGCAGGCTGGGAGCTTCTGTAAACTCCCCCACGCGGACTCA---
CCTCCGCGCGGAGC-------------------AGGCCCGCCGGCGGCCCC----
CCCCAACTCTTTGTTTTTAT----AGTATCTCTTCTGAGGA-ACCCAAA----
AAATGAATCAAAACTTTCAACAACGGATCTCTTGGTTCTGGCATCGATGAAGAACGCAGCGAAATGCGAT
ACGTAATGTGAATTGCAGAATTCAGTGAATCATCGAATCTTTGAACGCACATTGCGCCCGCTGGTATTCC
AGCGGGCATGCCTGTTCGAGCGTCATTTCAACCCTC----AAGCTCTG----------------------
---CTTGGTGTTGGGGCCCT--
GCCGGCCCTGCCGACCCCCTTCTCCGGGGGGCGGACTAGGAAGGCAGGCCCTGAAATACAGTGGCGGGCT
CGCTAGGA---CTCCGAGCGTAGTAGTTT--------TACCACCTCGCTA--
TGGACGAACTAGCGGTGCCC-TGCCGTAAAAAAACCCCCTATTTCTG---
AAAAGTTGACCTCGGATCAGGTAGGAATACCCGCTGAACTTAA---------------------------
----

--------------GAAGGTATGTGACAATATTTCCGTGT---GTTTCAATACAACCCC--
GCGAGTTTCATACCGCCAACA--CCACGCCGTAACGTT---GTCGCGGCTTATTTGGTCCG-
CCTGCCCATCCACCCATTTTTT--CGGCGGGGCGCACACTTCGAA-TTTTGGTGCGGGG-
TTTGATCACCTC-TATCACT-GATAAGCGTGACCCACAACG--CTGCCCTCCCTTACA------------
------TCCTTCACACTT-------------------------C----AATTGGCAAGACCG-------
ATGACGCTAATAA-
AATTGTTACAACAGCCGCTGAACTCGGTAAGGGTTCCTTCAAGTACGCATGGGTCCTTGACAAGCTCAAG

GCCGAGCGTGAGCGTGGTATCACCATCGATATTGCTCTGTGGAAGTTCGAGACTCCCAAGTACTATGTCA
CCGTCATTGGTATGTTGCAATCGCCTTTCTCCCTGTCATTACTGAAAAAACACGAGCTAACTGC------
----
AGTCGAATCTAGACGCTCCCGGTCACCGTGACTTCATCAAGAACATGCTTTCTGGTGCGTACTCGACCCC
TCAC-----CACTTCACGAATCGA----
AGCGACGTCGCGATGCCACGACATTCAAACTGATCCCCATTCTAGGCAAACCATCTCTGGCGAGCATGGC
CTCGACAGCAATGGCGTGTACGTA-------CTTGTCATCC-TACAACTGCG-----TGTC---
ACAGCTTAGATACTGACCG-
AAGCTCAGCTACAACGGTACCTCGGAGCTCCAGCTCGAGCGTATGAACGTCTACTTCAACGAGGTATGTT
CGCAG-------AGATT-----------
CCCGTCCACGTCTCCGGGCGCGAATTGCTACTATCGCCAAAGC--TCC-TCTAACGCGCA--
CCATTTCGAGTAGGCCTCCGGCAACAAATATGTTCCTCGCGCCGTTCTTGTCGATCTCGAGCCCGGTACC
ATGGACGCCGTGCGCGCCGGTCCTTTCGGTCAGCTGTTCCGTCCCGACAACTTCGTTTTCGGCCAGTCCG
GTGCTGGCAACAACTGGGCCAAGGG
>Pseudoplagiostoma_bambooensis_SAUCC_1206-4
CGAGTGAAGCGGCAACAGCTCAAATTTGAAATCTGGCTTCG--
GCCCGAGTTGTAATTTGCAGAGGATGCTTCTGGCGCGGTGCCTTCCGAGTTCCCTGGAACGGGACGCCAT
AGAGGGTGAGAGCCCCGTATGGATGGACACCAAGCCTGTGTGAAGCTCCTTCAACGAGTCGAGTAGTTTG
GGAATGCTGCTCTAAATGGGAGGTAAATCTCTTCTAAAGCTAAATATAGGCCAGAGACCGATAGCGCACA
AGTAGAGTGATCGAAAGATGAAAAGCACCTTGAAAAGGGGGTTAAATAGTACGTGAAATTGTTGAAAGGG
AAGCGCTTGTGACCAGACTTGTGCCAGGCGGCTCATCCGGTGTTCTCACCGGTGCACTCCGCCTG-
GCTCAGGCCAGCATCGGTTTTCGCGGGGGGACAAGAACGGTGGGAACGTGGCTC-CTCTCGGG-
GAGTGTTATAGCCCGCCGTACGATACCCTCGCGGGGACCGAGGTTCGCGCATCT----
GCAAGGATGCTGGCGTAATGGTCACCAGCGACCCGTCTTGAAACACGGACCAAGGAGTCGTCCATTAGAG
CGAGCGTTTGGGTGTCAAACCCGCACGCGTAATGAAAGTGAAAT-----
TAGGTGAGAGCTTCGGCGCATCATCGACCGATCCTGATGTTCTCGGATGGATTTGAGTAAGAGTTTTAAC
GGACGGACCCGAAAGACAGTGAACTATGCTTGTATAGGGTGAAGCCAGAGGAAACTCTGGTGGAGGCTCG
CAGCGGTTCTGACGTGCAAATCGATCGTCAAATATGAGCATGGGGGCGAAAGACTAATCGAACTGTCTAG
TATCTCCGTTGGTGAACCAGCGGAGGGATCATTGCTGGAA-GCGTCTCACGGCGCACCCAG---AT--
ACCCTTTGTGAACC-ATACC--
TCTCTGTTGCCTCGGCGCAGGCTGGGAGCTTCTGTAAACTCCCCCACGCGGACTCA---
CCTCCGCGCGGAGC-------------------AGGCCCGCCGGCGGCCCAA---CCAAACCTCTTG-
TTTTTAC----AGTGTCTCCTCTGAGTT-ACAAACAC---
AAATGAATCAAAACTTTCAACAACGGATCTCTTGGTTCTGGCATCGATGAAGAACGCAGCGAAATGCGAT
AAGTAATGTGAATTGCAGAATTCAGTGAATCATCGAATCTTTGAACGCACATTGCGCCCGCTGGTATTCC
AGCGGGCATGCCTGTTCGAGCGTCATTTCACCCCTC----CAGCTCCG----------------------
---CTGGGTGTTGGGGCACT--GCCG--------CCTCCCTCCGGGGAG--------------
AGGCAGGCCCTGAAATACAGTGGCGGGCTCGCTAAGA---CTCTGAGCGTAGTAGTT---------
TTACACCTCGTTC--TGGACGTGTTAGCGGTGCTCTTGCCGTAAAA--CCCCCCAATTTTCTG--
AAAAGTTGACCTCGGATCAGGTAGGAATACCCGCTGAACTTAA---------------------------
----

--------------GAAGGTATGTGAAAACATTTCAAAAT---CATTCAATCCAGCATC--
ATGAAATCCCCTCATGAAAAAGATGGGCCCGAGAATTCTGTGTGGTGGCCTATTTTGCTTG---
TGCTCATACACTCAATTTTT---GGCGGAGCGCACATTTTGGA-TTTTGGTGCGGGG-
TTTGATCACCTC-TATCTGT-
GATAAGCGTGACCCACCACGAGCAATCCCAACTTGTACCATCATCTCGTCTGCCCCATGTTATATGCGC-
-------CCTC-----TTTTCTC----AATTGGCTATTTT--------ACGATGCTAACTC-A---
ATCATTACAGCTGCTGAGCTCGGCAAGGGTTCCTTCAAGTACGCCTGGGTCCTTGACAAGCTCAAGGCCG
AGCGTGAGCGTGGTATCACCATCGACATTGCTCTGTGGAAGTTCGAGACTCCCAAGTACTATGTCACCGT
CATTGGTAAGTCGAATTCACTGTTCTGTTCG------CGGTTGAACCAGCAGCTAACTGT----------
----
GTCTACTTGCAGACGCTCCCGGTCACCGTGACTTCATCAAGAACATGCTTTCTGGTGCGTACTCGA--
CCACCA-----CATTCCTCATATCGG----
CGCGACATAACGATATCACGACATTGGTACTGACTTCAACCACAGGCAGACCATCTCTGGCGAGCACGGC
CTTGACAGCAATGGCGTGTACGTA-------CCTGCCATTC-TTCCATCGCG-----TGTT---
ATCGTAGTCATACTGACCG-
AAGCTCAGCTACAATGGTACCTCCGAGCTCCAGCTCGAGCGTATGAACGTCTACTTCAATGAGGTGAGTA
GAAA----------GGAC------CTCTGTCACTGTCGCC-
TAGGGCGCACATCGCCACTACTGCTGAGCT--TGT-
CCTGACGCGTTGCTTCTTTGGTCTAGGCTTCCGGCAACAAGTATGTTCCCCGCGCCGTCCTCGTCGATCT
CGAGCCCGGTACCATGGACGCCGTCCGTGCCGGTCCCTTCGGCCAGCTTTTCCGTCCCGACAACTTCGTC
TTCGGCCAGTCCGGTGCTGGCAACAACTGGGCCAAGGG
>Pseudoplagiostoma_bambooensis_SAUCC_1206-6
CGAGTGAAGCGGCAACAGCTCAAATTTGAAATCTGGCTTCG--
GCCCGAGTTGTAATTTGCAGAGGATGCTTCTGGCGCGGTGCCTTCCGAGTTCCCTGGAACGGGACGCCAT
AGAGGGTGAGAGCCCCGTATGGATGGACACCAAGCCTGTGTGAAGCTCCTTCAACGAGTCGAGTAGTTTG
GGAATGCTGCTCTAAATGGGAGGTAAATCTCTTCTAAAGCTAAATATAGGCCAGAGACCGATAGCGCACA
AGTAGAGTGATCGAAAGATGAAAAGCACCTTGAAAAGGGGGTTAAATAGTACGTGAAATTGTTGAAAGGG
AAGCGCTTGTGACCAGACTTGTGCCAGGCGGCTCATCCGGTGTTCTCACCGGTGCACTCCGCCTG-
GCTCAGGCCAGCATCGGTTTTCGCGGGGGGACAAGAACGGTGGGAACGTGGCTC-CTCTCGGG-
GAGTGTTATAGCCCGCCGTACGATACCCTCGCGGGGACCGAGGTTCGCGCATCT----
GCAAGGATGCTGGCGTAATGGTCACCAGCGACCCGTCTTGAAACACGGACCAAGGAGTCGTCCATTAGAG
CGAGCGTTTGGGTGTCAAACCCGCACGCGTAATGAAAGTGAAAT-----
TAGGTGAGAGCTTCGGCGCATCATCGACCGATCCTGATGTTCTCGGATGGATTTGAGTAAGAGTTTTAAC
GGACGGACCCGAAAGACAGTGAACTATGCTTGTATAGGGTGAAGCCAGAGGAAACTCTGGTGGAGGCTCG
CAGCGGTTCTGACGTGCAAATCGATCGTCAAATATGAGCATGGGGGCGAAAGACTAATCGAACTGTCTAG
TATCTCCGTTGGTGAACCAGCGGAGGGATCATTGCTGGAA-GCGTCTCACGGCGCACCCAG---AT--
ACCCTTTGTGAACC-ATACC--
TCTCTGTTGCCTCGGCGCAGGCTGGGAGCTTCTGTAAACTCCCCCACGCGGACTCA---
CCTCCGCGCGGAGC-------------------AGGCCCGCCGGCGGCCCAA---CCAAACCTCTTG-

TTTTTAC----AGTGTCTCCTCTGAGTT-ACAAACAC---
AAATGAATCAAAACTTTCAACAACGGATCTCTTGGTTCTGGCATCGATGAAGAACGCAGCGAAATGCGAT
AAGTAATGTGAATTGCAGAATTCAGTGAATCATCGAATCTTTGAACGCACATTGCGCCCGCTGGTATTCC
AGCGGGCATGCCTGTTCGAGCGTCATTTCACCCCTC----CAGCTCCG----------------------
---CTGGGTGTTGGGGCACT--GCCG--------CCTCCCTCCGGGGAG--------------
AGGCAGGCCCTGAAATACAGTGGCGGGCTCGCTAAGA---CTCTGAGCGTAGTAGTT---------
TTACACCTCGTTC--TGGACGTGTTAGCGGTGCTCTTGCCGTAAAA--CCCCCCAATTTTCTG--
AAAAGTTGACCTCGGATCAGGTAGGAATACCCGCTGAACTTAA---------------------------
----

--------------GAAGGTATGTGAAAACATTTCAAAAT---CATTCAATCCAGCATC--
ATGAAATCCCCTCATGAAAAAGATGGGCCCGAGAATTCTGTGTGGTGGCCTATTTTGCTTG---
TGCTCATACACTCAATTTTT---GGCGGAGCGCACATTTTGGA-TTTTGGTGCGGGG-
TTTGATCACCTC-TATCTGT-
GATAAGCGTGACCCACCACGAGCAATCCCAACTTGTACCATCATCTCGTCTGCCCCATGTTATATGCGC-
-------CCTC-----TTTTCTC----AATTGGCTATTTT--------ACGATGCTAACTC-A---
ATCATTACAGCTGCTGAGCTCGGCAAGGGTTCCTTCAAGTACGCCTGGGTCCTTGACAAGCTCAAGGCCG
AGCGTGAGCGTGGTATCACCATCGACATTGCTCTGTGGAAGTTCGAGACTCCCAAGTACTATGTCACCGT
CATTGGTAAGTCGAATTCACTGTTCTGTTCG------CGGTTGAACCAGCAGCTAACTGT----------
----
GTCTACTTGCAGACGCTCCCGGTCACCGTGACTTCATCAAGAACATGCTTTCTGGTGCGTACTCGA--
CCACCA-----CATTCCTCATATCGG----
CGCGACATAACGATATCACGACATTGGTACTGACTTCAACCACAGGCAGACCATCTCTGGCGAGCACGGC
CTTGACAGCAATGGCGTGTACGTA-------CCTGCCATTC-TTCCATCGCG-----TGTT---
ATCGTAGTCATACTGACCG-
AAGCTCAGCTACAATGGTACCTCCGAGCTCCAGCTCGAGCGTATGAACGTCTACTTCAATGAGGTGAGTA
GAAA----------GGAC------CTCTGTCACTGTCGCC-
TAGGGCGCACATCGCCACTACTGCTGAGCT--TGT-
CCTGACGCGTTGCTTCTTTGGTCTAGGCTTCCGGCAACAAGTATGTTCCCCGCGCCGTCCTCGTCGATCT
CGAGCCCGGTACCATGGACGCCGTCCGTGCCGGTCCCTTCGGCCAGCTTTTCCGTCCCGACAACTTCGTC
TTCGGCCAGTCCGGTGCTGGCAACAACTGGGCCAAGGG
